# Acidic nanoparticles protect against α‐synuclein‐induced neurodegeneration through the restoration of lysosomal function

**DOI:** 10.1111/acel.13584

**Published:** 2022-03-23

**Authors:** Marie‐Laure Arotcarena, Federico N. Soria, Anthony Cunha, Evelyne Doudnikoff, Geoffrey Prévot, Jonathan Daniel, Mireille Blanchard‐Desce, Philippe Barthélémy, Erwan Bezard, Sylvie Crauste‐Manciet, Benjamin Dehay

**Affiliations:** ^1^ Univ. Bordeaux CNRS IMN UMR 5293 Bordeaux France; ^2^ Achucarro Basque Center for Neuroscience Dpto. Neurociencias Universidad del País Vasco (UPV/EHU) Leioa Spain; ^3^ Université de Bordeaux INSERM U1212 CNRS UMR 5320 ARNA ARN: Régulations Naturelle et Artificielle ChemBioPharm Bordeaux France; ^4^ Université de Bordeaux Institut des Sciences Moléculaires CNRS UMR 5255 Talence France; ^5^ Biomedical Engineering and Imaging Institute Icahn School of Medicine at Mount Sinai New York New York USA

**Keywords:** acidic nanoparticles, alpha‐synuclein, neurodegeneration, therapeutics, in vivo, lysosomal restoration, Parkinson's disease

## Abstract

Parkinson's disease (PD) is an age‐related neurodegenerative disorder characterized by the loss of dopaminergic neurons in the substantia nigra, associated with the accumulation of misfolded α‐synuclein and lysosomal impairment, two events deemed interconnected. Protein aggregation is linked to defects in degradation systems such as the autophagy‐lysosomal pathway, while lysosomal dysfunction is partly related to compromised acidification. We have recently proven that acidic nanoparticles (aNPs) can re‐acidify lysosomes and ameliorate neurotoxin‐mediated dopaminergic neurodegeneration in mice. However, no lysosome‐targeted approach has yet been tested in synucleinopathy models in vivo. Here, we show that aNPs increase α‐synuclein degradation through enhancing lysosomal activity in vitro. We further demonstrate in vivo that aNPs protect nigral dopaminergic neurons from cell death, ameliorate α‐synuclein pathology, and restore lysosomal function in mice injected with PD patient‐derived Lewy body extracts carrying toxic α‐synuclein aggregates. Our results support lysosomal re‐acidification as a disease‐modifying strategy for the treatment of PD and other age‐related proteinopathies.

## Graphical abstract

We demonstrated that acidic nanoparticles, after intracerebral injection into a α‐synuclein‐based mouse model of PD, attenuated PD‐related neurodegeneration and synucleinopathy by mechanisms involving a rescue of compromised lysosomes. This study demonstrated that strategies enhancing or restoring lysosomal‐mediated degradation thus appear as tantalizing neuroprotective/disease‐modifying therapeutic strategies and would be of major therapeutic interest for PD.

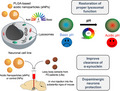

**Abbreviations**
AL: autophagolysosome
ALP: autophagy‐lysosomal pathway
aNPs: acidic nanoparticles
AP: autophagosome
BafA: bafilomycin A_1_
CMA: chaperone‐mediated autophagy
LB: Lewy Body
LC3: MAP1LC3B, microtubule‐associated protein 1 light chain 3 β
LMP: lysosomal membrane permeabilization
NPs: fluorescent organic nanoparticles
PD: Parkinson's disease
PLGA: poly(D,L‐lactide‐co‐glycolide)
ROI: region of interest
SNpc: substantia nigra *pars compacta*
TH: tyrosine hydroxylase
UPS: ubiquitin‐proteasome system
α‐syn: α‐synuclein

## 1 INTRODUCTION

Parkinson's disease (PD) is an age‐related, chronically progressive and incurable neurodegenerative disorder carrying great socioeconomic burden and impairing the elderly life of millions (Tysnes & Storstein, 2017). So far, the most consistent risk factor for developing PD is increasing age. PD is characterized by the progressive loss of dopaminergic neurons in the substantia nigra *pars compacta* (SNpc), which translates into the disruption of basal ganglia signaling and subsequent motor and cognitive deficiency. While the pathogenic mechanisms leading to PD are still not fully understood, a wealth of information points at a prominent role of α‐synuclein (α‐syn) (Dehay et al., 2015), an abundant neuronal protein involved in synaptic transmission (Logan et al., 2017). While α‐syn exists typically as an unfolded monomer, it acquires a toxic conformation and aggregate over the years in PD and related synucleinopathies (i.e., pathologies characterized by an accumulation of misfolded α‐syn), materializing as intracellular inclusions termed Lewy bodies (LB), often found in postmortem PD brains (Spillantini et al., 1997).

In addition, damaged and misfolded proteins accumulate during the aging process, when protein clearance systems might become less efficient, as illustrated by a decreased activity of both proteasomal and autophagic degradation pathways (Kaushik & Cuervo, 2015). The combination of aging and PD pathological changes could lead to a cellular stress condition that interferes with intracellular clearance pathways, favors α‐syn aggregation, and contributes to α‐syn‐mediated toxicity.

Lysosomal dysfunction is a significant event in proteinopathies (Nixon, 2013) recently associated with the two main neuropathological hallmarks of PD: dopaminergic neuronal cell death and accumulation of misfolded α‐syn (Dehay et al., 2013; Wallings et al., 2019). Impairment of lysosomal function, which is often associated with compromised acidification (Bagh et al., 2017; Dehay et al., 2012; Song et al., 2020), leads to defective protein and metabolite processing, leading to congestion of intracellular clearance systems. Since intracellular α‐syn species undergo degradation by either the ubiquitin–proteasome system (UPS) (Bennett et al., 1999) or the autophagy‐lysosomal pathway (ALP) (Cuervo et al., 2004), strategies enhancing these protein quality control machineries emerge as an attractive approach to reduce critical levels of toxic α‐syn (Dehay et al., 2016).

The re‐acidification of lysosomes has been suggested as a viable strategy in animal models of lysosomal storage disorders (Folts et al., 2016), which share risk‐factor genes and common pathogenic mechanisms with PD (Fraldi et al., 2016). Biocompatible poly(D,L‐lactide‐co‐glycolide) (PLGA) nanoparticles are state‐of‐the‐art tools for drug delivery in vivo, particularly useful in disease models since they can be internalized by targeted cells, including neurons (Cunha et al., 2021; Sahay et al., 2010). By exploiting the acidic properties of PLGA, rather than its functionality as a nanocarrier, we and others have demonstrated that these acidic nanoparticles (aNPs) can restore lysosomal acidification defects in a pathological context with a direct effect on the disease outcome (Baltazar et al., 2012; Bourdenx et al., 2016; Brouillard et al., 2021; Guha et al., 2014; Lee et al., 2015; Prevot et al., 2018; Senturk et al., 2019; van Veen et al., 2020; Xue et al., 2014). Nonetheless, while aNPs proved to be neuroprotective in vitro and in vivo in lysosomal‐impairment models related to PD, it is yet to be determined whether aNPs‐mediated lysosomal improvement affects α‐syn degradation, and therefore, α‐syn pathology.

Here, we investigate whether aNPs could ameliorate PD‐related phenotypes in mice injected with PD patient‐derived LB extracts carrying toxic α‐syn aggregates (Bourdenx et al., 2020; Recasens et al., 2014; Soria et al., 2020). We show that aNPs injection in vivo can prevent α‐syn‐triggered dopaminergic neurodegeneration, reduces α‐syn pathology and restores lysosomal function in the SNpc of these mice while enhancing α‐syn degradation and the ALP in neurons in vitro. These results confirm the therapeutic potential of aNPs and highlight lysosomal restoration as a disease‐modifying strategy for treating PD and other age‐related proteinopathies.

## 2 RESULTS

### 2.1 aNPs restore lysosomal function and integrity in vitro

**FIGURE 1:**
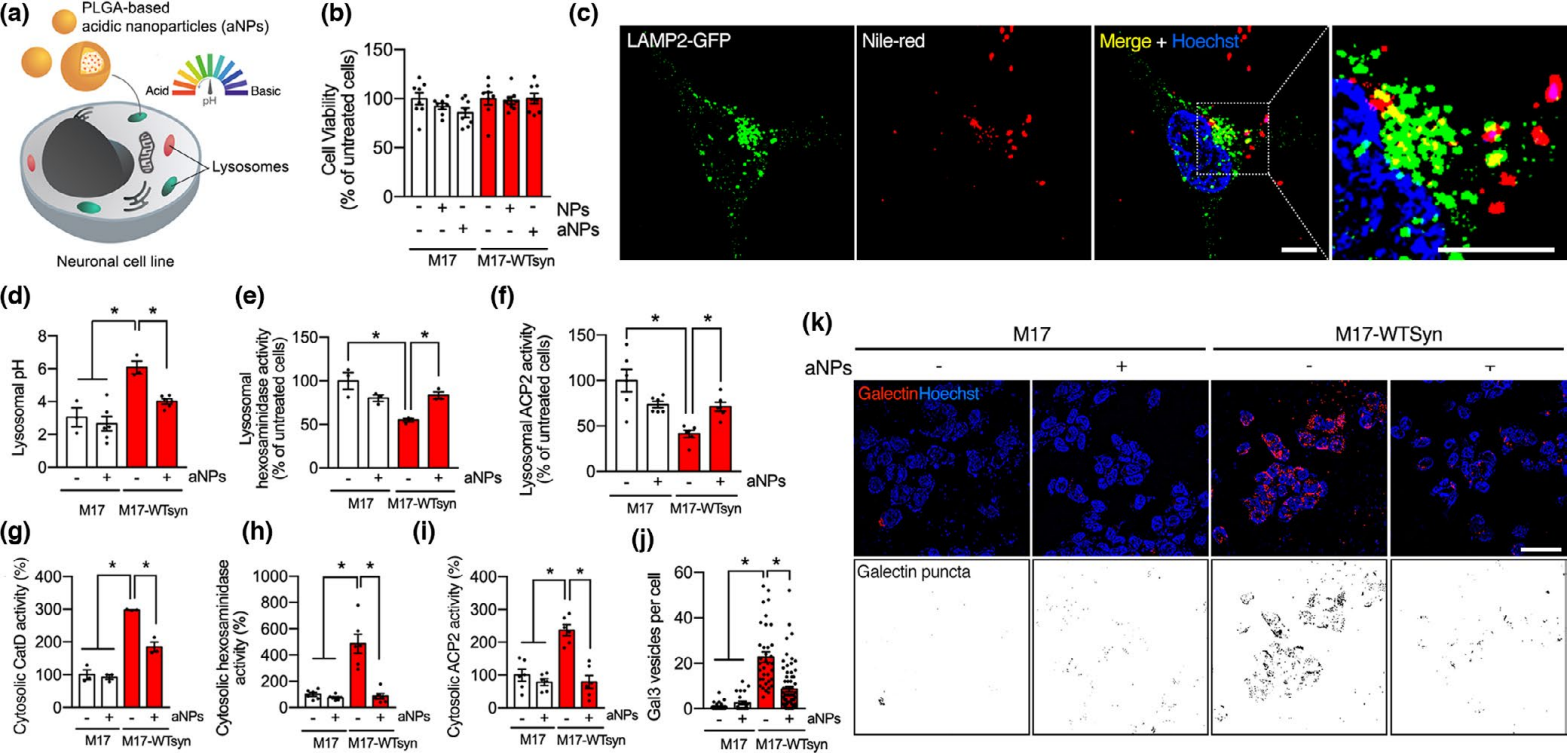
Acidic nanoparticles rescue lysosomal activity and integrity. (a) Schematic representation of in vitro delivery of aNPs into M17 cells. (b) Cell viability expressed as a percentage of untreated human dopaminergic neuroblastoma‐derived M17 cells and M17 cells overexpressing human WT α‐syn (M17‐WTsyn), treated with either nonacidic nanoparticles (NPs) or acidic nanoparticles (aNPs) for 24 h at 1 µg/ml. (Each dot represents an independent sample, *n* = 8). (c) Immunofluorescence images and insets of M17 cells treated with Nile Red‐loaded aNPs (1 µg/ml) and immunostained with LAMP2 as a marker of the lysosomal compartment at 24 h post‐incubation. Scale bar: 10 µm. (d) Lysosomal pH values as measured ratiometrically using LysoSensor Yellow/Blue DND‐160 in M17 and M17‐WTsyn cells treated with NPs (−) or aNPs (+) for 24 h (1 µg/ml). Each dot represents an independent sample. *n* = 3–6 per group (e, f) Lysosomal β‐hexosaminidase (e) and acid phosphatase precursor 2 (ACP2) (f) activity assay expressed as a percentage of NPs‐treated M17 in M17 and M17‐WTsyn cells treated with NPs (−) or aNPs (+) for 24 h (1 µg/ml). Each dot represents an independent sample. *n* = 3–6 per group. (g–i) Cytosolic β‐hexosaminidase (g), acid phosphatase precursor 2 (ACP2) (h), and cathepsin D (CatD) (i) activity assay expressed as a percentage of NPs‐treated M17 cells in M17 and M17‐WTsyn cells treated with NPs (−) or aNPs (+) for 24 h (1 µg/ml). Each dot represents an independent sample. *n* = 3–6 per group. (j, k) Quantification (j) and illustrative images (k) of Gal3‐positive dots per cell in M17 and M17‐WTsyn cells transfected with mCherry‐LGALS3/galectin‐3 (Gal3) probe (red, the nucleus in blue) for 24 h and treated with NPs (−) or aNPs (+) for 24 h (1 µg/ml). The lower panel of (k) constitutes a binarized representation of the mCherry channel. *n* = 23–67 cells per condition. Scale bar: 10 µm. All data are expressed as mean ± SEM. Two‐way ANOVA and Tukey's post hoc test. *p < 0.05 [(b) *F*(2, 42) = 1.70, *p* = 0.19; (d) *F*(3, 14) = 12.80, *p* = 0.0003; (e) *F*(1, 20) = 15.49, *p* = 0.0008; (f) *F*(1, 8) = 18.45, *p* = 0.0026; (g) *F*(1, 8) = 22.89, *p* = 0.0014; (h) *F*(1, 20) = 23.59, *p* < 0.0001; (i) *F*(1, 20) = 17.05, *p* = 0.0005; (j) *F*(1, 168) = 29.10, *p* < 0.0001]

We used aNPs to explore the effect of re‐acidifying lysosomes in an α‐syn context (Figure 1a). aNPs adopt a spherical shape with a mean diameter of 149.8 ± 0.9 nm (Figure S1), and zeta potential values of −41.5 ± 0.8 mV, as previously reported (Bourdenx et al., 2016). We have previously shown that BE(2)‐M17 neuroblastoma cells are suitable neuronal cell lines that internalize aNPs into lysosomes through the endocytic pathway (Bourdenx et al., 2016) and that these aNPs can re‐acidify defective lysosomes to basal levels and rescue impaired lysosomal function in BE (2)‐M17 cells stably depleted of lysosomal type 5 P‐type ATPase (ATP13A2), a PD cellular model (Bourdenx et al., 2016; Prevot et al., 2018). To precisely mimic increased levels of α‐syn found in pathological neurons, we then employed BE(2)‐M17 cells overexpressing human wild‐type α‐syn (M17‐WTsyn) (Baptista et al., 2003; Bisaglia et al., 2010). Fluorescent organic nanoparticles (NPs) are stable and water‐soluble particles obtained by nanoprecipitation of hydrophobic dyes. These soft NPs, which are PLGA‐free (i.e., no acidic behavior), were used as control for aNPs. In vitro cell viability assay confirmed that both types of nanoparticles do not induce cell death compared with untreated cells (Figure 1b) (Table S1 features all raw data). We confirmed that aNPs were successfully uptaken by every M17 and M17‐WTSyn cell at 24 h posttreatment (Figure S2A) and ultimately delivered to lysosomes, as evidenced by colocalization between the lysosomal marker LAMP2 and the Nile Red‐loaded aNPs (Figure 1c), where 10% to 15% of lysosomes are aNPs‐positive in M17‐WTSyn and M17 cells, respectively. Confocal optical sectioning and orthogonal projections of the resulting z‐stacks confirmed the colocalization between another lysosomal marker LAMP1 and aNPs 24 h after treatment (Figure S2B). Additional 3D reconstruction from z‐stack, where aNPs are found within LAMP1‐positive puncta, can be observed (Movie S1), a similar result reminiscent of our previous study (Bourdenx et al., 2016).

To determine whether wild‐type α‐syn overexpression may impact the intraluminal pH of lysosomes, we measured lysosomal pH with LysoSensor Yellow/Blue DND‐160. We observed that lysosomal pH acidification is compromised specifically in M17‐WTsyn (Figure 1d) in agreement with a recent study (Nascimento et al., 2020). Also, in vitro assays analyzing the activity of two lysosomal enzymes (i.e., ACP2 [acid phosphatase 2] and HEX/β‐hexosaminidase) in lysosomal fractions of M17‐WTsyn cells confirmed a markedly reduced proteolytic activity of these enzymes relative to control cells (Figure 1e,f). Treatment with aNPs allowed to rescue both lysosomal pH alkalization (Figure 1d) and proteolytic activity of lysosomal enzymes (Figure 1e,f) in M17‐WTsyn cells. These results indicate that wild‐type α‐syn overexpression impairs proper lysosomal acidification and proteolytic activity, which can be rescued by aNPs treatment.

Finally, we explored whether lysosomal integrity or stability was affected through lysosomal membrane permeabilization (LMP), a distinctive feature of lysosomal impairment (Boya & Kroemer, 2008). We measured the ectopic activity of three lysosomal‐resident proteases, cathepsin‐D (CatD), β‐hexosaminidase, and acid phosphatase 2 (ACP2) in lysosomal‐free cytosolic fractions from M17 and M17‐WTsyn cells. Compared with control cells, α‐syn overexpression triggered LMP as evidenced by a 3 to 5‐fold increase in the cytosolic activity of CatD (Figure 1g), β‐hexosaminidase (Figure 1h), and ACP2 (Figure 1i) in M17‐WTsyn cells, which were all rescued by aNPs treatment as measured in lysosome‐free cytosolic fractions (Figure 1g–i), suggesting a direct beneficial effect of aNPs on lysosomal membrane integrity in M17‐WTsyn cells. Further supporting the occurrence of LMP in M17‐WTsyn cells (Burbidge et al., 2021) and the impact of aNPs, we measured galectin puncta at leaky lysosomes as a highly sensitive assay for LMP. Accordingly, M17‐WTsyn cells exhibited increased Gal3‐positive vesicles per cell, using a mCherry‐LGALS3/galectin‐3 construct, which was entirely abolished by aNPs treatment (Figure 1j,k). These results corroborate the feasibility of restoring LMP by aNPs treatment. Overall, these results indicate that aNPs can restore a proper lysosomal function and integrity in cells overexpressing wild‐type α‐syn.

### 2.2 aNPs increase α‐syn degradation through ALP induction

**FIGURE 2:**
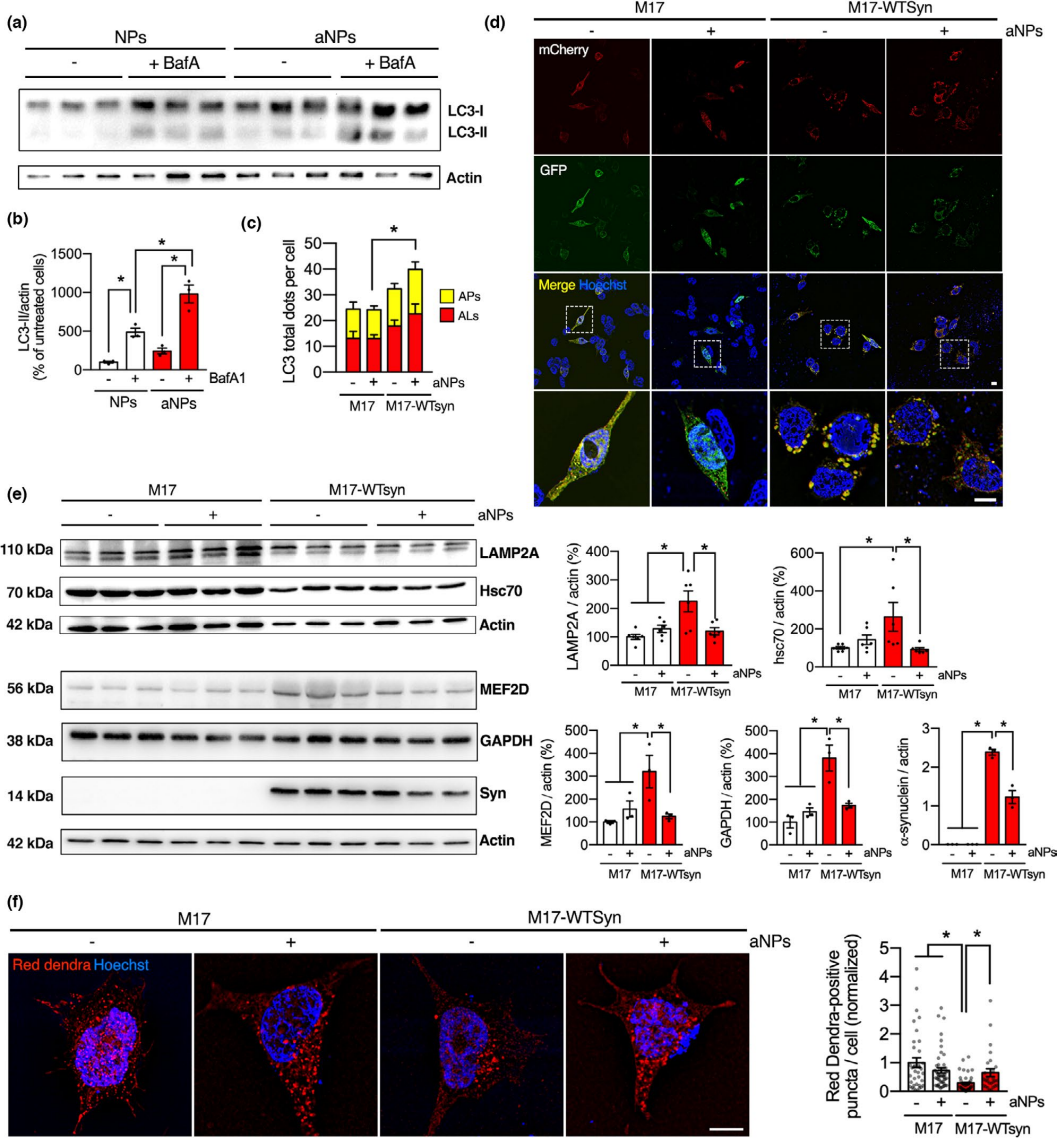
Acidic nanoparticles increase α‐syn degradation through induction of the autophagy‐lysosomal pathway. (a, b) Representative images (a) and immunoblot quantification (b) of LC3 protein levels normalized by actin in M17‐WTsyn cells treated with NPs or aNPs for 24 h (1 µg/ml) and co‐treated (+) or not (−) with BafilomycinA1 (BafA) at 500 nM for 1 h before the end of NPs/aNPs treatment. Each dot represents an independent sample. *n* = 3 per group. (c–d) Quantification (c) and illustrative images (d) of LC3‐positive dots per cell in M17 and M17‐WTsyn cells transfected with a mCherry‐GFP‐LC3 plasmid for 24 h and treated with NPs (−) or aNPs (+) for 24 h (1 µg/ml). Scale bar: 10 µm. (e) Representative images *(left)* and immunoblot quantifications *(right)* of LAMP2A, Hsc70, α‐synuclein, GAPDH, and MEF2D protein levels normalized by actin in M17 and M17‐WTsyn cells treated with NPs or aNPs for 24 h (1 µg/ml). Each dot represents an independent sample. *n* = 3–6 per group. Note: Although the cellular growth rate was similar both for control and α‐synuclein overexpressing cells, the presence of WT α‐synuclein had cytotoxic effects with a decrease in viability from 89% to 66% (WT) (Bisaglia et al., 2010), as evidenced here by the differences in actin levels. (f) Illustrative images *(left)* and quantification *(right)* of red Dendra‐positive dots per cell in M17 and M17‐WTsyn cells infected with a KFERQ‐PS‐Dendra lentivirus for 48 h and treated with NPs (−) or aNPs (+) for 24 h (1 µg/ml). Scale bar: 10 µm. APs: autophagosomes; ALs: autophagolysosomes. All data are expressed as mean ± SEM. Two‐way ANOVA and Tukey's (a–e) or Kruskal‐Wallis (f) post hoc test. *p < 0.05. [(b) *F*(1, 8) = 7.099, *p* = 0.0286; (c) *F*(3, 164) = 4.586, *p* = 0.0041; (e) *LAMP2A F*(1, 20) = 9.890, *p* = 0.0051/*Hsc70 F*(1, 20) = 6.989, *p* = 0.0156/*MEF2D F*(1, 8) = 9.907, *p* = 0.0137/*GAPDH F*(1, 8) = 15.24, *p* = 0.0045/*α*‐*synuclein F*(1, 8) = 40.69, *p* = 0.0002 (f) *F*(1, 148) = 7.095, *p* = 0.0086]

While α‐syn can be cleared by UPS, the main pathway for its degradation appears to be mediated by the lysosome (Webb et al., 2003). Because α‐syn can be degraded by both macroautophagy (Vogiatzi et al., 2008) and chaperone‐mediated autophagy (CMA) (Cuervo et al., 2004), we next explored whether aNPs could impact ALP, a system burdened by increased α‐syn load (Arotcarena et al., 2019; Martinez‐Vicente et al., 2008; Nascimento et al., 2020; Tang et al., 2021). To assess the impact of lysosomal re‐acidification by aNPs, we examined the autophagy flux in cells overexpressing α‐syn. M17‐WTSyn cells were exposed to a concomitant treatment of aNPs and Bafilomycin A1 (BafA), an inhibitor of lysosomal acidification (Figure 2a,b). As expected, the levels of MAP1LC3B/LC3B (microtubule‐associated protein 1 light chain 3 *β*)‐II (LC3‐II) were increased in both the control and aNPs‐treated cells in the presence of BafA (Figure 2a,b). However, the BafA + aNPs combined group exhibited a higher level of LC3‐II than the one in the aNPs or BafA alone‐treated groups (Figure 2a,b). These results suggest that aNPs significantly induce autophagy flux in M17‐WTSyn cells. To further validate this observation, M17 and M17‐WTsyn cells were transfected with a plasmid carrying the dynamic tandem fluorescent reporter mCherry‐GFP‐LC3 (Castillo et al., 2013) prior to aNPs treatment. Here, the pH‐sensitive GFP signal (Figure 2c,d, green) is quenched by the lysosome's acidic environment, whereas the mCherry signal (Figure 2c,d, red) is pH‐insensitive. Thus, autophagosomes (AP, not fused to lysosomes) are marked by both GFP and mCherry (yellow dots), whereas autophagolysosomes (AL, fused to lysosomes) are characterized only by mCherry (red dots). In M17‐WTSyn cells, we confirmed an accumulation of the two different types of vacuoles (red and yellow dots) and notably in the vacuole volume of yellow dots. In aNPs‐treated M17‐WTsyn cells, we observed a significant increase in AL number as shown by higher mCherry fluorescence intensity (Figure 2c,d). Quantification of yellow puncta in aNPs‐treated M17‐WTsyn cells showed a considerable increment, indicating an increase in autophagosome numbers (Figure 2c,d). These results suggest that aNPs significantly induce macroautophagy activity in these cells.

We next decided to evaluate the effect of aNPs treatment on CMA activity. Immunoblot analysis revealed accumulation of crucial players in CMA, LAMP2A, and Hsc70, as well as undegraded CMA substrates, α‐syn, GAPDH, and MEF2D (Aniento et al., 1993; Yang et al., 2009) in M17‐WTsyn cells (Figure 2e). Interestingly, aNPs decreased α‐syn levels to 50% in M17‐WTsyn cells and restored LAMP2A, Hsc70, GAPDH, and MEF2D proteins to control levels (Figure 2e), indicating that the degradation process inside the lysosome is rescued. To further corroborate aNPs‐mediated recovery of CMA‐dependent proteolysis, we then measured CMA activity dynamically in M17 and M17‐WTsyn cells with a lentivirus expressing the photoswitchable CMA‐targeted fluorescent substrate KFERQ‐PS‐Dendra (Koga et al., 2011). Upon 405 nm light exposure, Dendra protein is modified to emit red fluorescence, making it possible to track its delivery to lysosomes over newly synthesized protein. CMA activity is detected as a change from diffuse (cytosolic) fluorescence to a punctate (lysosomal) pattern. We observed that M17 cells exhibited a low cytosolic signal and some Dendra‐positive fluorescent puncta, reflecting basal CMA activity (Figure 2f). However, we observed a significantly reduced number of Dendra‐positive puncta throughout the cytoplasm in M17‐WTsyn cells, consistent with reduced CMA activity (i.e., the KFERQ‐PS‐Dendra reporter is not being correctly degraded by CMA and accumulates in the cytosol). When cells were treated 24 h with aNPs, we found a significant increase in Dendra‐positive puncta in M17‐WTsyn cells (Figure 2f), indicating restoration of CMA activity. These results confirm that aNP‐mediated re‐acidification of defective lysosomes in M17‐WTSyn cells promotes restoration of the ALP (i.e., improving α‐syn degradation).

### 2.3 aNPs prevent α‐syn‐induced neuronal loss and synucleinopathy in vivo

We have recently shown that aNPs treatment is neuroprotective against 1‐methyl‐4‐phenyl‐1,2,3,6‐tetrahydropyridine (MPTP) intoxication in mice (Bourdenx et al., 2016). While the MPTP mouse is a widely used animal model of parkinsonism, it does not recapitulate the progressive dopaminergic neuronal loss and α‐syn pathology concerning PD's natural progression. Therefore, we aimed to extend this observation and explore the translational potential of such an innovative strategy in a more clinically relevant model of PD related to α‐syn‐induced dopaminergic neurodegeneration. We thereby investigated this hypothesis via intracerebral injection of low doses (pg/µl) of α‐syn containing LB extracts purified from the SNpc of PD brains, previously shown to promote α‐syn pathology and progressive dopaminergic degeneration in wild‐type mice and nonhuman primates (Bourdenx et al., 2020; Recasens et al., 2014).

**FIGURE 3:**
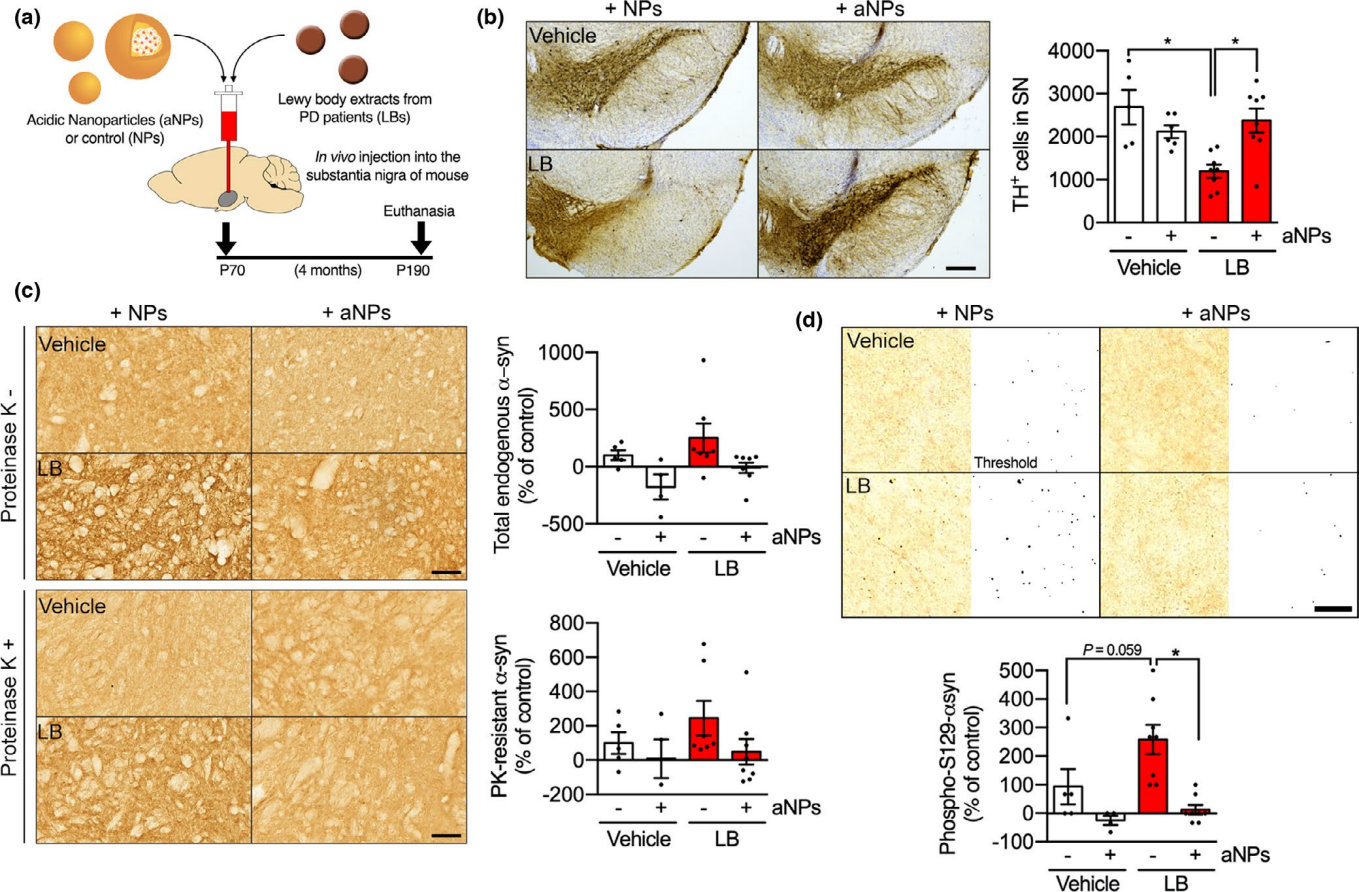
Acidic nanoparticles prevent α‐syn‐induced neurodegeneration and synucleinopathy in mice. (a) Schematic and timeline of the experimental procedure. (b) Representative images *(left)* and quantification *(right)* of Tyrosine Hydroxylase (TH)‐ positive neurons by stereological counting in the substantia nigra (SN) of control (Vehicle) and LB‐injected mice 4 months after injection of nonacidic nanoparticles (NPs) or acidic nanoparticles (aNPs). Scale bar: 500 µm. (c) Representative images (*left*) and quantification (*right*) of α‐synuclein immunostaining without (*up*) or with (*bottom*) proteinase K (PK) treatment using syn1 antibody in the SN of control (Vehicle) and LB‐injected mice 4 months after injection of nonacidic nanoparticles (NPs) or acidic nanoparticles (aNPs). Scale bar: 200 µm. (d) Representative (*up*, *left*), binarized (*up*, *right*) images and quantification (*bottom*) of S129‐phosphorylated α‐synuclein immunostaining using EP1536Y antibody in the SN of control (Vehicle) and LB‐injected mice 4 months after nonacidic nanoparticles (NPs) or acidic nanoparticles (aNPs). Each dot represents an animal. *n* = 5–8 per group. All data are expressed as mean ± SEM. Two‐way ANOVA and Tukey's post hoc test. *p < 0.05. [(b) *F*(1, 23) = 12.04, *p* = 0.0021; (c) *Top F*(1, 20) = 0.007819, *p* = 0.9304/*Bottom F*(1, 20) = 0.3123, *p* = 0.5825; (d) *F*(1, 21) = 16.51, *p* = 0.0006]

To demonstrate the feasibility and therapeutic potential of this strategy in this PD mouse model, we next assessed at different time‐points whether we were still able to detect Nile Red‐loaded aNPs within tyrosine hydroxylase (TH)‐positive cells, a marker for dopaminergic neurons, without evident cytotoxicity at 24 h, 48 h, 1 and 4 month after intracerebral injection (Figure S3). We further confirmed aNPs delivery into lysosomes of nigral dopaminergic cells in vivo by the co‐presence of Nile Red‐loaded aNPs with lysosomal marker LAMP2 in TH‐positive cells at 24 h, 48 h, 1 and 4 month postinjection (Figure S3). We injected LB fractions into the SNpc of adult mice, concomitantly with aNPs or control NPs (vehicle solution was used as a control for LB injection), and performed the full histopathological analysis 4‐months after surgery (Figure 3a). At 4 months postinjection, stereological cell counts of TH‐positive neurons in mouse SNpc revealed that aNPs were able to reduce dopaminergic cell loss to 20% instead of ~50% of α‐syn‐induced neurodegeneration typically observed in this model (Bourdenx et al., 2020; Recasens et al., 2014; Soria et al., 2020) (Figure 3b). This was associated with an attenuation of TH‐positive fibers loss in these animals at the striatal dopaminergic terminals (Figure S4A).

Progressive neurodegeneration courses with mild neuroinflammation, and in our model, most glial reactivity takes place early after LB injection (Soria et al., 2020). This prompted us to inject aNPs alongside LB instead of a later time‐point, since in our experience, extensive glial activation occurs upon two consecutive intracerebral injections at the same site. At 4 months postinjection, we detected no increase in glial reactivity neither in the SNpc nor the striatum, as evidenced by comparable staining of astrocytic marker GFAP or microglia/macrophage protein Iba1 (Figure S4B,C). This suggests that any neuroinflammation that might be triggered by LB + aNPs injection is resolved by the end of the experiment and shows that aNPs‐induced neuroprotection is independent of neuroinflammatory processes.

We next explored whether the neuroprotection associated with aNPs treatment observed in the LB mouse model was also accompanied by a reduction in α‐syn pathology. Total and proteinase K (PK)‐resistant (i.e., aggregated) α‐syn was seemingly decreased in the presence of aNPs, although the trend was not significant neither in the SNpc (Figure 3c) nor in the striatum (Figure S4d). Since our model is not based on viral vector‐driven α‐syn overexpression, and α‐syn concentration in the inoculum is five orders of magnitude lower than that in other α‐syn seeding models (Luk et al., 2012), PK‐resistant aggregates are less common (Soria et al., 2020). Consequently, we performed immunohistochemical investigations in the SNpc with a phospho‐specific Ser129 α‐syn antibody as a second more sensitive readout of pathological α‐syn (Fujiwara et al., 2002). Phospho‐S129‐α‐syn levels were increased in LB‐injected mice compared with control, indicating the presence of toxic α‐syn, and were almost completely restored to basal values by aNPs (Figure 3d). These results suggest a dramatic reduction in α‐syn‐induced neurodegeneration and pathology through aNPs administration.

To gain mechanistic insight for the reduced α‐syn pathology conveyed by aNPs treatment at 4 months postinjection, we assessed the effects of aNPs on injected human α‐ syn uptake at short term (24h, 48h) and middle term (1 month) after LB injection. We previously demonstrated that exogenously injected LB fractions containing human α‐syn are internalized by TH‐positive neurons within 24 h (Recasens et al., 2014; Soria et al., 2020), and consequently induce the formation of de novo endogenous murine α‐syn aggregates. Here, we found no difference in human α‐syn‐positive immunostaining present in nigral dopaminergic neurons of LB + aNPs mice compared with LB mice at 24 h and 48 h postinjection, suggesting that aNPs do not prevent the initial uptake of human‐α‐syn that triggers the endogenous pathological process (Figure S5). In agreement with previous observations in this model (Recasens et al., 2014; Soria et al., 2020), we did not find human‐α‐syn at 1‐month postinjection in both groups (Figure S5). However, histological analysis of endogenous α‐syn‐positive immunostaining at 1 month postinjection revealed a significant decrease of total murine α‐syn in the LB + aNPs group compared with the LB group, further suggesting that aNPs protect from endogenous α‐synuclein accumulation even at 1 month postinjection (Figure S6A,B). Moreover, the presence of α‐syn protein into aNP‐positive lysosomes of SNpc TH‐positive neurons in LB‐injected mice at 1 month postinjection suggested that α‐syn protein is uptaken by aNPs‐positive lysosomes for its degradation (Figure S6C).

### 2.4 aNPs induce lysosomal recovery in dopaminergic neurons in vivo

**FIGURE 4:**
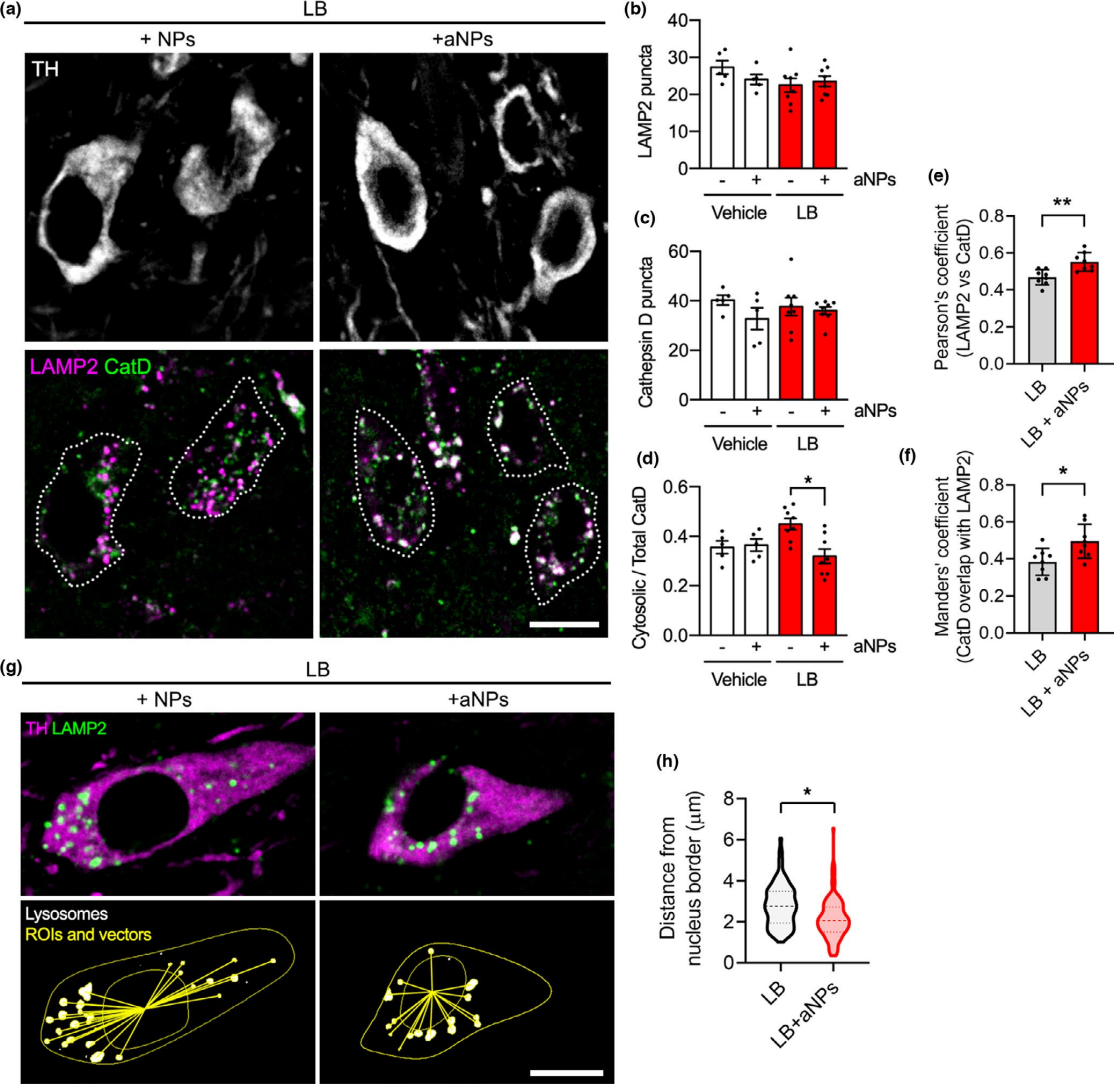
Acidic nanoparticles induce lysosomal recovery in dopaminergic neurons in LB‐injected mice. (a) Representative confocal micrographs of triple immunostaining for dopaminergic marker tyrosine hydroxylase (TH), lysosomal marker (LAMP2), and lysosomal protease Cathepsin D (CatD), in the substantia nigra (SN) of LB‐injected mice 4 months after nonacidic nanoparticles (NPs) or acidic nanoparticles (aNPs) injections. Scale bar = 10 µm. (b–c) Quantification of lysosomal and CatD puncta in TH‐positive neurons from the SN of control (Vehicle) and LB‐injected mice 4 months after nonacidic nanoparticles (NPs) or acidic nanoparticles (aNPs) injections. Mean ± SEM. Each dot represents an animal. *n* = 5–8 per group. Two‐way ANOVA and Tukey's post hoc test. (d) Lysosomal membrane permeabilization represented as the ratio of cytosolic CatD over total CatD, quantified from colocalization images as in (a, *right panel*). Mean ± SEM. Each dot represents an animal. *n* = 5–8 per group. Two‐way ANOVA and Tukey's post hoc test. *p < 0.05. [(b) *F*(1, 22) = 1.505, *p* = 0.2329; (c) *F*(1, 22) = 0.8829, *p* = 0.3576; (d) *F*(1, 22) = 6.257, *p* = 0.0203] (e, f) Colocalization analysis of LAMP2 and CatD signals, expressed as Pearson's correlation coefficient (e) and Manders’ coefficient to represent the fraction of CatD overlapping with LAMP2 (f). Unpaired two‐tailed *t*‐test. *p < 0.05. **p < 0.01. (g, h) Image segmentation analysis (g) and quantification (h) to examine lysosomal distance to the nucleus. LAMP2‐positive lysosomes were segmented, and the distance to the border of the nuclear ROI (traced in the TH channel) was calculated for each lysosome. Scale bar = 10 µm. Violin plot with median and quartiles, *n* = 8 animals (10 cells per animal), Nested two‐tailed *t*‐test, *p < 0.05

Finally, we investigated whether aNPs are able to rescue α‐syn‐induced lysosomal dysfunction in vivo similarly to the in vitro results reported above. To this end, we examined the cellular distribution of lysosomal markers (LAMP2 and CatD puncta) and the occurrence of LMP (i.e., the cytosolic release of CatD and other hydrolases from the lysosomal lumen to the cytosol) in SNpc TH‐positive neurons by confocal fluorescence microscopy and segmentation analysis at 4 months postinjection (Figure 4a). We detected no changes in the number of lysosomes through LAMP2 (Figure 4b) or CatD (Figure 4c) puncta quantification, suggesting that neither LBs nor aNPs alter lysosomal biogenesis in these cells at 4 months. However, we observed a peak of LAMP2‐positive puncta at 48 h postinjection (Figure S7) while this effect was no longer present neither at 1 or 4 months postinjection. These results suggest that aNPs may enhance the lysosomal function in an early step, which lasts over time. By subtracting the fraction of CatD in colocalization with lysosomal marker LAMP2 (i.e., lysosomal CatD), we detected increased cytosolic levels of CatD in LB‐injected mice, which were entirely restored by aNPs (Figure 4d). We confirmed this result by analyzing Pearson's and Manders’ coefficients for colocalization for these two signals. We observed increased colocalization of LAMP2 and CatD in the LB + aNPs group (Figure 4e), and an increased fraction of CatD overlapping with LAMP2 (Figure 4f), indicating more CatD inside lysosomes when LB mice were treated with aNPs. We further confirmed that CatD not colocalizing with LAMP2 belongs to the cytosolic compartment, as evidenced by the absence of co‐staining with the endoplasmic reticulum marker PDI or the Golgi protein 58K (Figure S8). This suggests that aNPs mitigate LMP, which is an indicator of sick lysosomes and has harmful effects on the cell (Boya & Kroemer, 2008).

Interestingly, it has been recently suggested that the subcellular localization of lysosomes determines their luminal pH, with peripheral lysosomes suffering from alkalinized pH and impaired proteolytic activity (Johnson et al., 2016). Thus, we evaluated lysosomal positioning in nigral TH‐positive neurons in vivo by segmentation analysis of confocal images at 4 months postinjection. Following cell segmentation into nuclear and cytoplasmic regions of interests (ROIs) and detection of LAMP2‐positive vesicles, we calculated the distance of each LAMP2‐positive lysosome to the nucleus border (Figure 4g). Strikingly, dopaminergic neurons in SNpc of LB + aNPs mice displayed lysosomes that were closer to the nucleus than in the LB group (Figure 4h), in agreement with recent in vitro studies (Johnson et al., 2016), and indirectly suggesting that a higher amount of lysosomes from aNPs‐treated LB mice presented acidic pH. Since lysosomal redistribution is a protective cellular mechanism used by the cell to cope with stress (Korolchuk et al., 2011), we hypothesize that the perinuclear localization of aNPs‐treated lysosomes would be a sign of overall cellular health.

Altogether, our results suggest that aNPs treatment in vivo is efficient to restore proper lysosomal function and integrity in nigral dopaminergic neurons of LB‐injected animals.

## 3 DISCUSSION

Here, we first confirmed that aNPs were able to restore proper lysosomal function and integrity through re‐acidification of the lysosomal lumen in wild‐type α‐syn overexpressing cells. We further showed in vitro that ALP‐mediated α‐syn degradation is enhanced through the restoration of lysosomal function induced by aNPs treatment. Remarkably, aNPs also restored in vivo lysosomal function, reduced α‐syn pathology, and ultimately protected dopaminergic neurons from α‐syn‐induced cell death in LB‐injected mice. Both in PD and synucleinopathy models based on α‐syn injection, it has been proposed that aggregated α‐syn transmits its misfolded conformation by recruiting α‐syn monomers (i.e., “seeding”) in a prion‐like manner, spreading pathology through neuroanatomically connected regions (Jucker & Walker, 2013). In our particular model, exogenously injected toxic α‐syn (from a human origin) is internalized by TH‐positive SNpc neurons within 24 and 48 h postinjection (Recasens et al., 2014; Soria et al., 2020), since subsequent de novo aggregates containing mostly endogenous (rodent) protein are produced. Since aNPs are found in the brain long after intraparenchymal injection (Bourdenx et al., 2016; Prevot et al., 2018), they likely boost degradation of endogenous, de novo aggregated, α‐syn rather than human α‐syn seeds. This is supported by the observation of decreased levels of endogenous α‐syn found in the LB + aNPs‐treated mice after 1 month, and by the observation that aNPs‐mediated decrease in phospho‐S129‐α‐syn at 4 months, which labels neurotoxic α‐syn before oligomerization (Chen & Feany, 2005), is more significant than the amelioration on PK‐resistant α‐syn labeling, which is a marker of dense aggregates.

We also demonstrated that aNPs‐mediated restoration of lysosomal activity in surviving TH neurons is a long‐term effect, present at 4 months postinjection. The strong influence on neurodegeneration would then be associated with increased partially α‐syn degradation and with the promotion of overall cellular health by improving protein clearance. This is supported by the decreased LMP process as well as an increased number of lysosomes relocalized to the perinuclear area observed in aNPs‐treated animals that indirectly illustrate a specific restoration of proper lysosomal function through re‐acidification. Even though both LBs and aNPs might be internalized by glial cells in the vicinity or by other neurons present in the SNpc, this lysosome‐boosting effect would be particularly beneficial to aged dopaminergic neurons, whose degradation systems are overstrained by higher metabolic demand and elevated oxidative stress (Trist et al., 2019). Furthermore, given that approximately 50% of TH‐positive neurons are lost after LB injection (compared with 20% in the LB + aNPs group), our observations on lysosomal health in these mice are likely underestimated since we have no data from dead neurons. Thus, the overall difference in lysosomal health (i.e., both LMP and lysosomal localization) between LB and LB + aNPs groups might be even more significant. Future developments of tools aimed to monitor lysosomes in vivo and in real time, plausibly by live imaging, will help to clarify this question.

In the context of neurodegenerative diseases associated with protein aggregation, such as PD, Alzheimer's disease, or amyotrophic lateral sclerosis, lysosomes are critical organelles essential for the clearance of amyloidogenic proteins. Thus, treatments aimed at improving lysosomal function, such as the method we present here, might prove useful for proteinopathies other than PD or lysosomal‐storage disorders. A similar approach recently used graphene nanoparticles to tackle the problem by impeding α‐syn fibrilization and disassembling aggregates, with comparable successful results regarding dopaminergic cell loss (Kim et al., 2018). pH‐responsive nanoparticles have also been used to target acidic organelles to treat chronic pain in rodents (Ramirez‐Garcia et al., 2019). Because of their organic nature, all these nanoparticles can potentially be tailored for delivery to specific cell types through chemical conjugation. Moreover, we have recently demonstrated that aNPs can be loaded into oil‐in‐water nanoemulsions to facilitate the crossing of the blood‐brain barrier and therefore enable systemic delivery (Prevot et al., 2018). Then, nanoparticle‐based approaches emerge as a therapeutic opportunity that would allow to address the origin of PD and similar proteinopathies rather than mitigate its effects (Cunha et al., 2021).

## 4 EXPERIMENTAL PROCEDURES

### 4.1 Preparation of acidic nanoparticles

The protocol was adapted from our previous study (Bourdenx et al., 2016). Acidic nanoparticles (aNPs) solutions were freshly prepared using the nanoprecipitation method. Briefly, 31 mg of Resomer^®^ RG 503H PLGA (Sigma‐Aldrich, 719870) and 1.0 mg of Nile Red (Sigma‐Aldrich, 19123) were dissolved in 3.1 ml of tetrahydrofuran (THF, Sigma‐Aldrich, 401757). 200 µl of this stock solution was quickly added to 20 ml of deionized water under sonication (10 W, 3 min). The resulting clear pink solution was then slowly concentrated by centrifugation using 10 kDa centrifugal filters (Merck Millipore, Amicon Ultra centrifugal Filter) for 21 min. at 5000 RPM (Centrifuge 5804 R, Eppendorf). Once the supernatant was collected, the resulting dark pink solutions were ready for use (typical concentration of PLGA ~1 mg/ml). As control conditions, fluorescent organic nanoparticles (NPs) were prepared following a similar procedure, 0.4 mg of hydrophobic Nile Red dye (Daniel et al., 2016) was dissolved in 1 ml of THF, then 200 µl of the resulting stock solution was added to 20 ml of deionized water under sonication (10 W, 3 min). The resulting red solutions are used without any further purification.

Physical characteristics of these NPs were assessed by DLS, Zetameter, and TEM. The hydrodynamic size of the NPs was measured using a DLS device from Malvern Instruments (Zetasizer Nano ZS). NPs were diluted at 1:1000 (v/v) and the mean size was determined on three independent measurements performed at 25°C. Zeta potential measurement was performed using Zetasizer Nano ZS coupled with Folded Capillary Cell (DTS1060) from Malvern Instruments. aNPs were then characterized using TEM, which was carried out using a HITACHI H7650 (HITACHI Ltd., Tokyo, Japon) electron microscope at 80 kV and a TALOS F200S (ThermoFisher Talos) electron microscope at 190 kV. Briefly, the copper grid coated with a carbon membrane was pretreated using the Glow discharge technique to yield a positively charged hydrophilic carbon surface to allow stronger interaction between the sample and the grid itself and thus easier imaging. One droplet of the nanoparticles (aNPs or NPs) aqueous suspension was deposited on the grid, and the excess liquid was dried off with a paper. A staining procedure using uranyl acetate was used to enhance the contrast. The nanoparticles were randomly and manually counted using the ImageJ program (using a circle selection).

### 4.2 Cell lines and cell viability assay

Human neuroblastoma cell lines BE(2)‐M17 (i.e., M17‐EV and M17‐WTSyn) were used as previously described (Baptista et al., 2003) and were provided by B. Wolozin (Boston University School of Medicine). Cells were maintained at 37°C in 5% CO_2_ in OPTIMEM (Thermo Fisher Scientific) plus 10% FBS supplemented with 1% streptomycin/penicillin (MilliporeSigma) and 500 µg/ml geneticin (G418) (Thermo Fischer Scientific). To assess cell viability, M17‐EV (i.e., M17 in the text) and M17‐WTsyn cell lines were plated in 96‐wells plate and treated with nonacidic nanoparticles (NPs) or acidic nanoparticles (aNPs) for 24 h at 1 µg/ml. Cell viability was then estimated by an MTT assay (ATCC, 30‐1010K) following the manufacturer's recommendations.

### 4.3 Lysosomal uptake of aNPs

To confirm the delivery of aNPs to lysosomes, M17 and M17‐WTsyn cell lines were plated on coverslips in a 12‐well plate. Cells were maintained for 24 h at 37°C in 5% CO_2_ before being treated with Nile Red‐loaded aNPs for 24 h at 1µg/ml. Cells coverslips were fixed at 4°C for 20 min using 4% paraformaldehyde. The fixed cells were washed three times with PBS1X for 5 min each time. Cells coverslips were permeabilized for 5 min into PBS–Saponine 0.02%, blocked in 4% goat serum/PBS for 1 h at RT, and incubated overnight at 4°C with mouse anti‐LAMP2 (SantaCruz, sc18822, 1:1000). Following incubation with primary antibody, cell coverslip was washed with PBS three times and incubated for 1 h at RT with a corresponding goat anti‐mouse IgG conjugated to AlexaFluor probe (Invitrogen, 1:400). Coverslips were finally stained with DAPI solution (Invitrogen) at 10 μM for 8 min before long washes in PBS1X and mounted onto slides using a mounting solution (Dako). Image acquisitions were made on a wide‐field Olympus Epifluorescent Microscope (BX3‐CBH) coupled with a Hamamatsu camera (ORCA‐Flash 4.0 LT). Images were deconvolved using the cellSens Dimension software. All image acquisitions and analyses were performed blinded to the researcher. In parallel, transient transfection experiment with LAMP1‐GFP was performed in M17 and M17‐WTSyn cells with Lipofectamine 2000 (Life Technologies, 11668019) following the manufacturer's recommendations. Cells were maintained for 24 h at 37°C in 5% CO_2_ before being treated with Nile Red‐loaded aNPs for 24 h at 1 µg/ml. Confocal images were obtained in a Leica TCS SP8 microscope. Image stacks (pixel size ~100 nm, z‐step 0.3 μm) were acquired with an 63× Plan Apo CS objective with oil immersion. Colocalization, fluorescence profiles, orthogonal projections, and maximal intensity projections were obtained with the ImageJ (NIH) distribution Fiji.

### 4.4 Lysosomal pH measurement

Quantification of lysosomal pH was determined using dextran conjugates LysoSensor Yellow/Blue DND‐160 (Life Technologies) and was performed as previously described (Dehay et al., 2012). Briefly, M17 and M17‐WTsyn cell lines were grown in their respective media and treated with Nile Red‐loaded aNPs for 24 h at 1 µg/ml. Cells were then trypsinized, harvested (1 × 10^6^ cells/ml), and loaded with 1 mg/ml of LysoSensor‐dextran for 1 h at 37°C with 5% CO_2_. The cells were then washed 3W in HBSS (Gibco, 14060) and aliquoted at 100 ml into a black 96‐well microplate. pH calibration was performed as previously described (Dehay et al., 2012). M17 and M17‐WTsyn cells were treated with 10 mM monensin (Sigma‐Aldrich) and 10 mM nigericin (Sigma‐Aldrich) in MES buffer (5 mM NaCl, 115 mM KCl, 1.3 mM MgSO_4_, 25 mM MES), with the pH adjusted to a range from 3.5 to 7.0. The samples were read in a FLUOstar Optima fluorimeter (BMG Labtech, Champigny sur Marne, France) with excitation at 355 nm. The ratio of emission 440/535 nm was then calculated for each sample. The pH values were determined from the standard linear curve generated via the pH calibration samples.

### 4.5 CTSD, HEX/β‐hexosaminidase, and ACP2 activity assay

The integrity of the lysosomal membrane in cultured cells was monitored by analyzing the presence of three lysosomal enzymes, namely, cathepsin D, HEX/β‐hexosaminidase, and acid phosphatase in the cytosol. M17 and M17‐WTsyn cell lines were plated in 12‐wells plate and treated with nonacidic (NPs) or acidic nanoparticles (aNPs) for 24 h at 1 µg/ml. CTSD activity was measured using a fluorometric CTSD activity assay kit (Abcam, ab65302) in accordance with the manufacturer's instructions. Fluorescence was measured on a FLUOstar Optima microplate analyzer (BMG Labtech). Phosphatase activity was assayed with the help of the acid phosphatase assay kit (CS0740; Sigma), according to the manufacturer's instructions, as well as the β‐hexosaminidase assay kit (CS0780; Sigma). To measure the cytosolic cathepsin D, acid phosphatase, and β‐hexosaminidase activity, cells were rinsed extensively with ice‐cold PBS and then removed by trypsinization in the homogenization buffer. The lysosomal fraction was isolated by differential sedimentation in sucrose homogenization buffer (0.25 M sucrose, 20 mM Hepes) as described in (Dehay et al., 2012). The cytosolic fractions were prepared by centrifugation of the supernatant of the light mitochondrial–lysosomal fraction at 100,000× *g* for 30 min, as extensively described in (Dehay et al., 2012). Prewarmed (37°C) substrate solution and reaction components were mixed in a 96‐well plate. Briefly, the β‐hexosaminidase activity was determined by the addition of 90 μl of assay buffer [0.09 M citrate buffer (pH4.8); 1 mM 4‐nitrophenyl N‐acetyl‐β‐D‐glucosaminide] to 10 μl of cell lysates. The acid phosphatase activity was determined by the addition of 50 μl of assay buffer [0.09 M citrate buffer (pH 4.8); 1 mM 4‐nitrophenyl phosphate] to 50 μl of cell lysates. For both assays, the reaction mixture was incubated at 37°C for 10 min and stopped by the addition of 200 μl of stop solution (0.5 N of NaOH). The absorbance was measured at 405 nm. CTSD activity was measured with 50 µl of samples and was incubated with reaction buffer supplied in the kit for 1 h at 37°C according to manufacturer's protocol. Fluorescence was measured (Ex/Em = 328/460 nm). Fold increases in protease activity were determined by comparing the relative fluorescence units (RFUs) against the levels of the controls. CTSD, acid phosphatase, and β‐hexosaminidase activity were measured in triplicate. We normalized the activity of lysosomal proteases by correcting the activity in the cytosol toward the activity in the lysosomal fractions.

### 4.6 Tandem fluorescent reporter mCherry‐GFP‐LC3 assay

To assess autophagy flux, the mCherry‐GFP‐LC3 probe was used as previously described (Castillo et al., 2017). Briefly, M17 and M17‐WTsyn cell lines were plated on coverslips in 12‐wells plate and were transfected with mCherry‐GFP‐LC3 plasmid at 1.6 µg DNA using PEI‐mediated transfection. Cells were maintained for 24 h at 37°C in 5% CO_2_ before being treated with nonacidic nanoparticles (NPs) or acidic nanoparticles (aNPs) for 24 h at 1 µg/ml. Cells coverslips were fixed at 4°C for 20 min using 4% paraformaldehyde. The fixed cells were washed three times with PBS1X for 5 min each time. Cells coverslips were finally stained with DAPI solution (Invitrogen) at 10 μM for 8 min before long washes in PBS1X. Coverslips were mounted onto slides using a mounting solution (Dako), and image acquisitions were made on a wide‐field Olympus Epifluorescent Microscope (BX3‐CBH) coupled with a Hamamatsu camera (ORCA‐Flash 4.0 LT). All image acquisitions and analyses were performed blinded to the researcher. Images were deconvolved using the cellSens Dimension software. Image analysis was performed in Fiji/ImageJ (Schindelin et al., 2012) using custom scripts (available upon request). For mCherry analysis, cells were segmented by manual cell selection. The mask was then applied to the mCherry channel, where red staining was quantified by automatic thresholding followed by binarization. Total staining was normalized per cell. A minimum of 20 cells were analyzed for each condition. Co‐localization was obtained from binary images of mCherry and GFP channels with the ImageCalculator plugin (function “AND”) and expressed as Yellow dots (autophagosome). Red dots correspond to the subtraction between Yellow dots and total mCherry staining (autophagolysosome).

### 4.7 Lysosomal galectin puncta assay

To assess the lysosomal galectin puncta assay, the mCherry‐LGALS3/galectin‐3 plasmid (kindly provided by Harald Wodrich's lab) was used as previously described (Aits et al., 2015). Briefly, M17 and M17‐WTsyn cell lines were plated on coverslips in 12‐wells plate and were transfected with mCherry‐LGALS3/galectin‐3 plasmid at 1.6 µg DNA using PEI‐mediated transfection. Cells were maintained for 24 h at 37°C in 5% CO_2_ before being treated with nonacidic nanoparticles (NPs) or acidic nanoparticles (aNPs) for 24 h at 1 µg/ml. Cells coverslips were fixed at 4°C for 20 min using 4% paraformaldehyde. The fixed cells were washed three times with PBS1X for 5 min each time. Cells coverslips were finally stained with DAPI solution (Invitrogen) at 10 μM for 8 min before long washes in PBS1X. Coverslips were mounted onto slides using a mounting solution (Dako), and image acquisitions were made on a wide‐field Olympus Epifluorescent Microscope (BX3‐CBH) coupled with a Hamamatsu camera (ORCA‐Flash 4.0 LT). All image acquisitions and analyses were performed blinded to the researcher. Images were deconvolved using the cellSens Dimension software. Image analysis was performed in Fiji/ImageJ (Schindelin et al., 2012). For mCherry analysis, cells were segmented by manual cell selection. The mask was then applied to the mCherry channel, where red staining was quantified by automatic thresholding followed by binarization. Total staining was normalized per cell, and a minimum of 20 cells were analyzed for each condition.

### 4.8 Assessment of CMA activity

To assess the CMA activity, a lentivirus expressing the photoactivable CMA‐specific fluorescent substrate KFERQ‐PS‐Dendra was used as previously described (Koga et al., 2011). Briefly, M17 and M17‐WTsyn cell lines were plated on coverslips in a 12‐well plate and were infected with 0.5 ml of lentivirus supernatant (kindly provided by Ana Marίa Cuervo's lab) using polybrene‐mediated infection for 48 h. Then, photoconversion of cells grown on coverslips was carried out with a 405/20 nm LED array for 3 min. Cells were then treated with nonacidic nanoparticles (NPs) or acidic nanoparticles (aNPs) at 1 µg/ml and maintained for 24 h at 37°C in 5% CO_2_. Cells coverslips were fixed at 4°C for 20 min using 4% paraformaldehyde. The fixed cells were washed three times with PBS1X for 5 min each time. Cells coverslips were finally stained with DAPI solution (Invitrogen) at 10 μM for 8 min before long washes in PBS1X. Coverslips were mounted onto slides using a mounting solution (Dako), and image acquisitions were made on a wide‐field Olympus Epifluorescent Microscope (BX3‐CBH) coupled with a Hamamatsu camera (ORCA‐Flash 4.0 LT). All image acquisitions and analyses were performed blinded to the researcher. Images were deconvolved using the cellSens Dimension software. Image analysis was performed in Fiji/ImageJ (Schindelin et al., 2012). For red Dendra analysis, cells were segmented by manual cell selection. The mask was then applied to the red channel, where red staining was quantified by automatic thresholding followed by binarization. Total staining was normalized per cell, and a minimum of 20 cells were analyzed for each condition.

### 4.9 In vitro immunoblotting assessment

M17 and M17‐WTsyn cell lines were plated in 12‐well plate and treated with nonacidic (NPs) or acidic nanoparticles (aNPs) for 24 h at 1 µg/ml. For the Bafilomycin A1 experiment, cells were co‐treated with 500 nM of BafilomycinA1 (Sigma) for 1 h at the last hour of NPs/aNPs treatment. Cells were washed and resuspended with cold phosphate‐buffered saline at 4°C and transferred to Eppendorf tubes. After a centrifugation step at 1300 *g* for 5 min, the supernatant was removed, and the cell pellet was lysed in 100 μl of Laemmli buffer (Tris‐HCl 25 mM, pH 6.8, glycerol 7.5%, SDS 1%, DTT 250 mM, and Bromophenol blue 0.05%). Western blots were run from 20 μl cell lysates separated by SDS‐PAGE and transferred to 0.2 μm nitrocellulose membrane (Bio‐Rad). Incubation of primary antibodies was performed overnight at 4°C with mouse anti‐α‐synuclein (1:1000, clone syn211, Thermo Scientific, MS1572), mouse anti‐MEF2D (1:1000; BD Transduction Laboratories, 610774), mouse anti‐GADPH (1:1000, clone 6C5, Abcam, 8245), rabbit anti‐LC3 (1:1000, Novus Biologicals, 100‐2220), rabbit anti‐LAMP2A (1:1000, Abcam, 18528), rat anti‐Hsc70 (1:1000, clone 1B5, Enzo Life Sciences, ADI‐SPA‐815). Mouse anti‐actin (1:2000, Millipore Sigma, A5441) was used to control equal loading. Appropriate secondary antibodies coupled to peroxidase were revealed using a Super Signal West Pico Chemiluminescent kit (Immobilon Western, Chemiluminescent HRP substrate, Millipore Sigma). Chemiluminescence images were acquired using the ChemiDoc + XRS system measurement (Bio‐Rad). Signals per lane were quantified using ImageJ, and a ratio of the signal on loading per sample was performed and used in statistical analyses.

### 4.10 Purification of Lewy bodies from human Parkinson's disease brains

Human SNpc was dissected from fresh frozen postmortem midbrain samples from three patients with sporadic Parkinson's Disease exhibiting conspicuous nigral LB pathology on neuropathological examination (mean age at death: 76 ± 2.2 years; frozen postmortem interval: 29.5 ± 4.91 h; GIE Neuro‐CEB BB‐0033‐00011). Tissue was homogenized in 9 vol (w/v) ice‐cold MSE buffer (10 mM MOPS/KOH, pH 7.4, 1 M sucrose, 1mM EGTA, and 1mM EDTA) with protease inhibitor cocktail (Complete Mini; Boehringer Mannheim) with 12 strokes of a motor‐driven glass/Teflon homogenizer. For LB purification, a sucrose step gradient was prepared by overlaying 2.2 M with 1.4 M and finally with 1.2 M sucrose in volume ratios of 3.5:8:8 (v/v). The homogenate was layered on the gradient and centrifuged at 160,000× *g* for 3 h using a SW32.1 rotor (Beckman). Twenty‐six fractions of 1500 μl were collected from each gradient from the top (fraction 1) to bottom (fraction 26) and analyzed for the presence of α‐synuclein aggregates by filter retardation assay, as previously described (Recasens et al., 2014). Further characterization of LB fractions was performed by immunofluorescence, α‐synuclein ELISA quantification, and electron microscopy as previously described (Bourdenx et al., 2020). For stereotactic injections, LB‐containing fractions from Parkinson's disease patients were mixed together in the same proportion (PD #1, fractions 19, 20, and 21; PD #2, fractions 19 and 20; PD #3, fraction 22). The amount of α‐syn in the LB fractions was quantified by human α‐syn ELISA kit (#KHB0061; Invitrogen/Life Technologies), corresponding to ~24 pg α‐synuclein per microliter of injected sample. In all cases, samples were bath‐sonicated for 5 min prior to in vivo injections.

### 4.11 Animals and surgical procedures

Forty‐eight wild‐type C57Bl/6J male mice (9 weeks old) (Charles River) received either 2 μl of either LB fractions or Vehicle (i.e., sucrose 2,2M), supplemented with 1 µl of either nonacidic (NPs) or acidic nanoparticles (aNPs) at 1 mg/ml by stereotactic delivery to the region immediately above the right SN (coordinates from bregma: AP: –2.9; L: 1,3; DV: –4.5) at a flow rate of 0.4 μl/min with a 30‐gauge Hamilton syringe coupled to a microinjection pump (World Precision Instruments). The pipette was left in place for 8 min after injection to avoid leakage. Animals were terminated after 24 h [Veh + aNPs: *n* = 2; LB + NPs: *n* = 2; LB + aNPs: *n* = 2], 48 h [Veh + aNPs: *n* = 2; LB + NPs: *n* = 2; LB + aNPs: *n* = 2], 1 month [Veh + aNPs: *n* = 3; LB + NPs: *n* = 3; LB + aNPs: *n* = 3], and 4 months [Veh + NPs: *n* = 5; Veh + aNPs: *n* = 6; LB + NPs: *n* = 8; LB + aNPs: *n* = 8]. The animals from the Veh + aNPs group received Nile Red‐loaded aNPs to follow the nanoparticles, whereas the other groups received clear aNPs for the need of future immunofluorescence co‐staining. Animals were perfused using saline solution followed by 4% paraformaldehyde fixation. Brains were then collected, immediately immersed in cold 4% paraformaldehyde for 24 h, then cryoprotected with 20% sucrose, and finally frozen by immersion in isopentane at −60°C before sectioning at 40 µm thickness.

### 4.12 Histopathological analysis

To assess the integrity of the nigrostriatal pathway, TH immunohistochemistry was performed on SNpc and striatal sections. Briefly, free‐floating sections from three representative levels of the striatum (anterior, medial, and posterior) and serial sections (1 of 4) corresponding to the whole SNpc were incubated with a rabbit monoclonal antibody raised against mouse TH (Abcam, EP1532Y, ab137869, 1:5000) for 1 night at room temperature and revealed with anti‐rabbit peroxidase EnVision^®^ system (DAKO, K400311) followed by DAB visualization. Sections were mounted on gelatinized slides, counterstained with 0.1% cresyl violet solution, dehydrated, and cover‐slipped, while striatal sections were mounted on gelatinized slides and cover‐slipped. The extent of the lesion in the striatum was quantified by optical density (OD). Sections were scanned in an Epson expression 10000XL high‐resolution scanner, and images were used in ImageJ open‐source software to compare the gray level in the putamen. TH‐positive SNpc cells were counted by stereology, blind with regard to the experimental condition, using a Leica DM6000B motorized microscope coupled with the Mercator software (Explora Nova). The SN was delineated for each slide, and probes for stereological counting were applied to the map obtained. Each TH‐positive cell with its nucleus included in the probe was counted. The optical fractionator method was finally used to estimate the total number of TH‐positive cells in the SNpc of each mouse hemisphere.

Synucleinopathy was assessed with a mouse monoclonal antibody raised against total α‐synuclein (BD Transduction Laboratories, clone 42, #610787, 1:1000) and against phosphorylated α‐synuclein (Abcam, EP1536Y, ab51253, 1:5000), as previously reported (Bourdenx et al., 2020; Recasens et al., 2014). Briefly, selected sections of one rostrocaudal level of SN and striatum were explicitly identified and incubated in the same well to allow direct comparison of immunostaining intensity. For pretreatment with proteinase K (PK), sections were incubated first with PK at 1 μg/ml in PBS for 10 min at room temperature before long sequential washes in distilled water and then in PBS. Sections were incubated overnight at room temperature with the aforementioned antibodies. The revelation was performed as described for TH staining. We measured the area of total α‐synuclein staining in SN and striatum with and without PK pretreatment to fully characterize the pattern of synucleinopathy. High‐resolution whole color slide images were acquired with a 3D Histech Panoramic Scanner at the 20× magnification, with five layers in extended mode. Each image was opened in Mercator Pro 7.12.3 software (Explora Nova), and all regions of interest were mapped. Brightness and contrast rules were applied to the RGB pictures to optimize details without any saturation of the image. The color thresholding tool was then used to select the threshold corresponding to the brown color revealed by the DAB staining. The threshold has been established on the basis of the staining intensity to detect the maximum of DAB staining. These parameters were applied to all measurements for each animal/staining. The area corresponding to the threshold defined was expressed as a ratio of the total area of each region of interest (ROI), normalized by the contralateral side for each animal and expressed as a percentage of the control group. In addition, phosphorylated α‐synuclein‐positive dots in SN were counted by stereology, similarly as with TH‐positive neurons. The optical fractionator method estimated the total number of phosphorylated α‐synuclein‐positive dots per μm² in the SN of each mouse.

At 1 month postinjection, induction of endogenous synucleinopathy was assessed in the SNpc using a specific rabbit monoclonal antibody raised against murine α‐synuclein (1:1000, clone D37A6, CST, 4179S). The immunohistochemical protocol was performed as described for TH staining. The level of α‐synuclein‐positive staining was quantified by optical density (OD).

Inflammation was measured with GFAP/S‐100 (DAKO, Z0334/Abnova, PAP11341) and Iba1 (Abcam, ab5076) antibodies. Nigral and striatal sections of all animals were incubated together overnight with a mix of rabbit antibodies raised against GFAP and S‐100 for the astroglial staining (respective dilutions 1:2000 and 1:1000) and with a rabbit anti‐Iba1 antibody for the microglial staining (dilution 1:1000) and revealed and processed as described above. GFAP‐positive astrocytic reaction or Iba1‐positive microglial reaction was estimated by area quantification at regional levels with Mercator Pro software (Explora Nova).

### 4.13 Immunofluorescence and image analysis

Tissue sections were permeabilized for 1 h in 4% donkey or goat serum/PBS blocking buffer containing 0.2% Saponine (Millipore Sigma) and incubated overnight at 4°C with the following primary antibodies diluted in a 1% donkey or goat serum/PBS buffer depending on the different needed combinations: rabbit anti‐TH (Abcam, EP1532Y, ab137869, 1:2000), mouse anti‐TH (Merck, clone LNC1, MAB318,1:2000), goat anti‐TH (Novus Biologicals, NB300‐110, 1:2000), rabbit anti‐CatD (Abcam, EPR3057Y,ab75852, 1:1000), rat anti–LAMP2 (Abcam, Abl93, ab25339, 1:1000), mouse anti‐human α‐synuclein (ThermoFisher Scientific, Syn211, 32–8100, 1:1000), rabbit anti‐mouse α‐synuclein (Cell Signaling Technology, D37A6, 4179, 1:1000), goat anti‐58K Golgi protein (Abcam, ab19072, 1:1000), mouse anti‐PDI (Enzo LifeSciences, clone 1D3, ADI‐SPA‐891, 1:1000). Tissue sections were washed in PBS and incubated for 2 h at room temperature with a combination of a corresponding donkey or goat anti‐species IgG conjugated with Alexa Fluor 350, 488, 594, and 647 (Invitrogen). Sections were washed with PBS and mounted in DAPI‐containing mounting media (Vectashield).

For illustrative images at 24 h, 48 h, 1‐ and 4‐month postinjection, image acquisitions were made on a wide‐field Olympus Epifluorescent Microscope (BX3‐CBH) coupled with a Hamamatsu camera (ORCA‐Flash 4.0 LT). All image acquisitions and analyses were performed blinded to the researcher. Images were deconvolved using the cellSens Dimension software.

For LAMP2 count and analysis in TH‐positive cells at 24 h, 48 h, and 1 month postinjection, image analysis was performed in Fiji/ImageJ. Cells were segmented by manual cell selection using the TH channel. The mask was then applied to the LAMP2 channel, where LAMP2 staining was quantified by automatic thresholding followed by binarization. Total staining was then normalized per cell.

For quantitative analysis at 4 month postinjection, images (pixel size = 80 nm) were acquired in sequential mode (to minimize channel crosstalk), with a 63X Plan Apo CS objective with oil immersion (numerical aperture = 1.4) in a Leica TCS SP8 confocal microscope. All image acquisitions and analyses were performed blinded to the researcher. Image analysis was performed in Fiji/ImageJ (Schindelin et al., 2012) using custom scripts (available at https://github.com/SoriaFN/Lysosome_analysis; Soria, 2021). For LAMP2/CatD analysis, three‐channel (TH/LAMP2/CatD) confocal images (pixel size = 80 nm) were denoised by difference of Gaussians (DoG) and segmented in the TH channel by manual thresholding. The TH channel‐based mask was then applied to LAMP2 and CatD channels, where lysosomal and CatD puncta were quantified by automatic thresholding followed by binarization (puncta smaller than 10 px were discarded). Total LAMP2 and CatD puncta were normalized per cell, and 10–20 cells were analyzed per animal. Colocalization was obtained from LAMP2 and CatD binary images with the ImageCalculator plugin (function “AND”). Further colocalization analysis was performed in a subset of LAMP2 and CatD images with the JACoP plugin (Bolte & Cordelieres, 2006) to estimate Pearson's correlation and Manders’ overlap coefficients. To estimate cytosolic CatD, LAMP2/CatD‐positive puncta was subtracted from total CatD to determine non‐lysosomal CatD fraction. For lysosomal distance analysis, cytoplasmic and nuclear ROIs were manually segmented using the TH channel of single z‐planes where the nucleus was clearly visible. To prevent a bias due to low cytoplasmic area and to ensure that the comparisons were made between cells with similar cytoplasmic ROIs, the analysis was subsequently performed only in cells where the ratio of cytoplasmic to nuclear ROI area was 2:1. Lysosomal puncta were segmented from the LAMP2 channel, and the distance from the nucleus center to the center of mass of each lysosomal ROI was calculated. Finally, to estimate the distance to the nucleus border, the average radius of an ellipse fitted to the nuclear ROI was subtracted from the distance to the nucleus center.

### 4.14 Statistics

Statistical analyses were performed with Prism 8 (GraphPad). Comparisons among means were performed by using two‐way ANOVA followed, if appropriate, by pairwise comparison between means by Tukey post hoc test. For colocalization analysis (Pearson's and Manders’ coefficient), unpaired two‐tailed t‐test was used. For the analysis of lysosomal distance to the nucleus, a nested t‐test was performed, comparing two treatments, in eight animals per treatment (mixed model). All values are expressed as the mean ± SEM unless specified otherwise in the figure legend. Statistical significance was set at *p* < 0.05.

### 4.15 Study approval

#### 4.15.1 Animals

Experiments were performed in accordance with the European Union directive of September 22, 2010 (2010/63/EU), on the protection of animals used for scientific purposes. The Institutional Animal Care and Ethical Committee of Bordeaux University (CE50, France) approved experiments accepted by the ministry under references APAFIS#8876−2016121452125 and APAFIS#24160.

#### 4.15.2 Human tissues

The samples were obtained from brains collected in a Brain Donation Program of the Brain Bank “GIE NeuroCEB” run by a consortium of Patients Associations: ARSEP (association for research on multiple sclerosis), CSC (cerebellar ataxias), France Alzheimer, and France Parkinson. The consent documents were signed by the patients themselves or their next of kin in their name, in accordance with the French Bioethical Laws. The Brain Bank GIE NeuroCEB (Bioresource Research Impact Factor number BB‐0033‐00011) has been declared at the Ministry of Higher Education and Research and has received approval to distribute samples (agreement AC‐2013‐1887).

## CONFLICT OF INTEREST

E.B. owns equity stake in Motac Holding Ltd. and receives consultancy payments from Motac Neuroscience Ltd. All other authors declare no competing interests.

## AUTHORS' CONTRIBUTION

BD isolated LB fractions, AC and JD prepared NPs and aNPs. FNS and MLA performed surgeries. MLA, FNS, ED, and GP performed histopathological analysis. MLA and BD carried out cell culture assays. FNS and MLA prepared the figures. FNS, MLA, and BD wrote the paper with input from all authors. BD, SC‐M, FNS, and GP conceptualized the study and designed experiments. MB‐D, PB, EB, SC‐M, and BD secured funding.

## Supporting information

Video S1

Table S1

Fig S1‐S8

## Data Availability

The original contributions presented in the study are included in the article, further inquiries can be directed to the corresponding author.
