# Supplementary material for: Acidic nanoparticles protect against α‐synuclein‐induced neurodegeneration through the restoration of lysosomal function
Source: Aging Cell. 2022 Mar 23;21(4):e13584. doi: 10.1111/acel.13584 (PMC9009122; doi:10.1111/acel.13584)
Supplement: Supplementary file 3 — Fig S1‐S8 [file ACEL-21-e13584-s003.docx]

**Supplemental information**

**
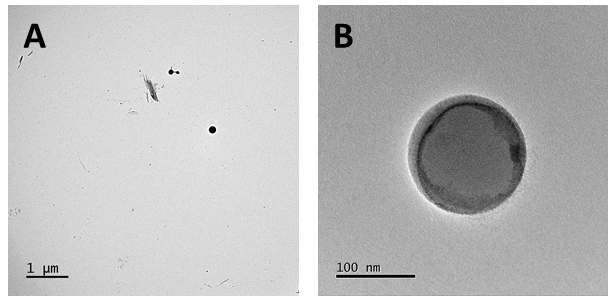
**

**Figure S1. TEM images of acidic nanoparticles.**  Transmission electron microscopy imaging of aNPs obtained using (**A**) a HITACHI H7650 (HITACHI Ltd., Tokyo, Japon) electron microscope at 80 kV and (**B**) a TALOS F200S (ThermoFisher Talos) electron microscope at 190 kV. Observation was made after negative staining with uranyl acetate.

**
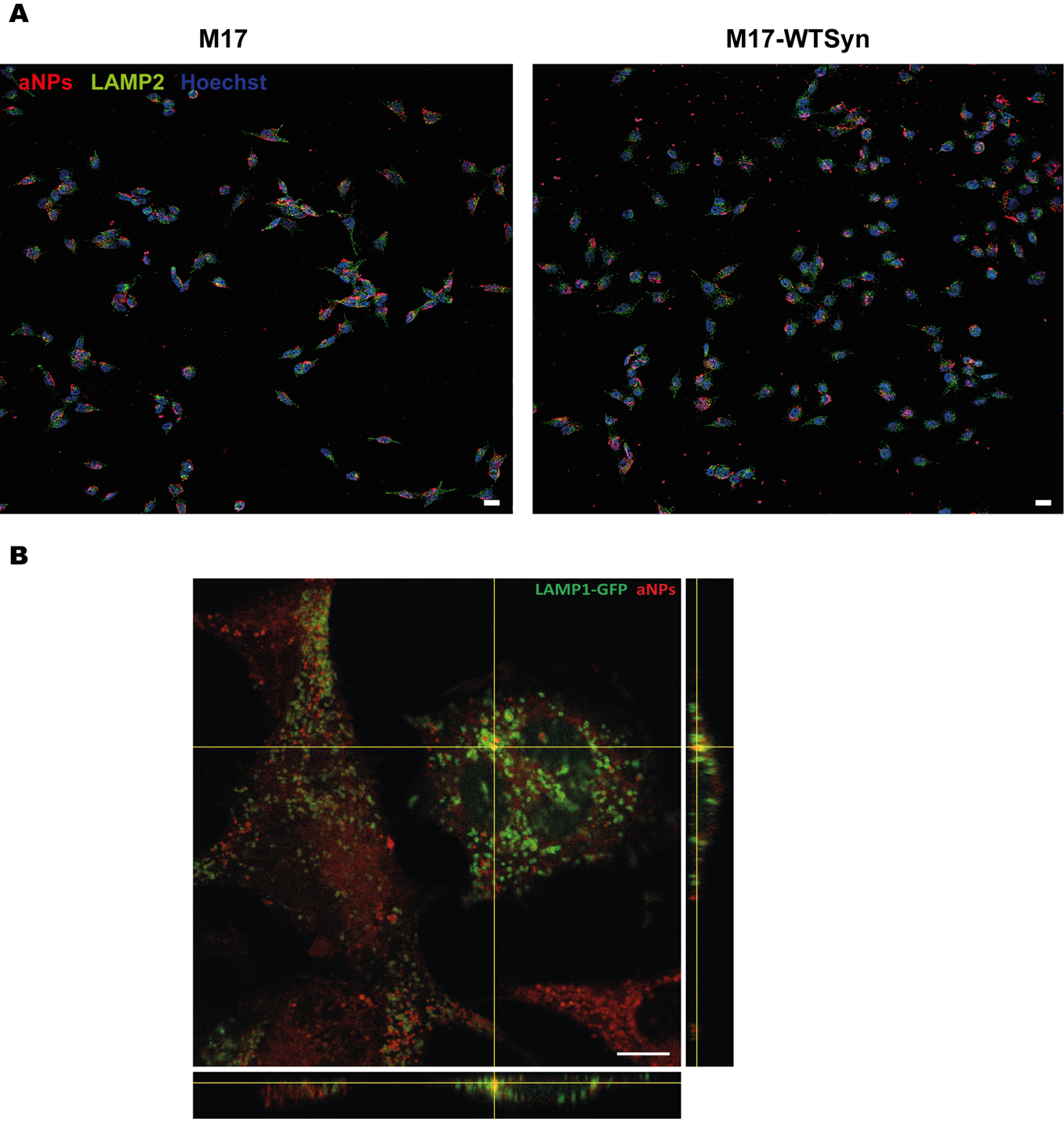
**

**Figure S2. Acidic nanoparticles are efficiently uptaken in the cells and detected in lysosomes.** **(A)** Representative immunofluorescent images of lysosomal marker LAMP2 *(green)* and Nile red-loaded aNPs *(red)* in M17 *(left)* and M17-WTSyn *(right)* cells at 24h post-treatment. Scale bar: 10µm. **(B)** Orthogonal projection of a confocal z-stack of in vitro immunofluorescence. Note the colocalization between acidic NPs (red) and lysosomal marker (LAMP1-GFP signal; green). Scale bar: 10 µm.


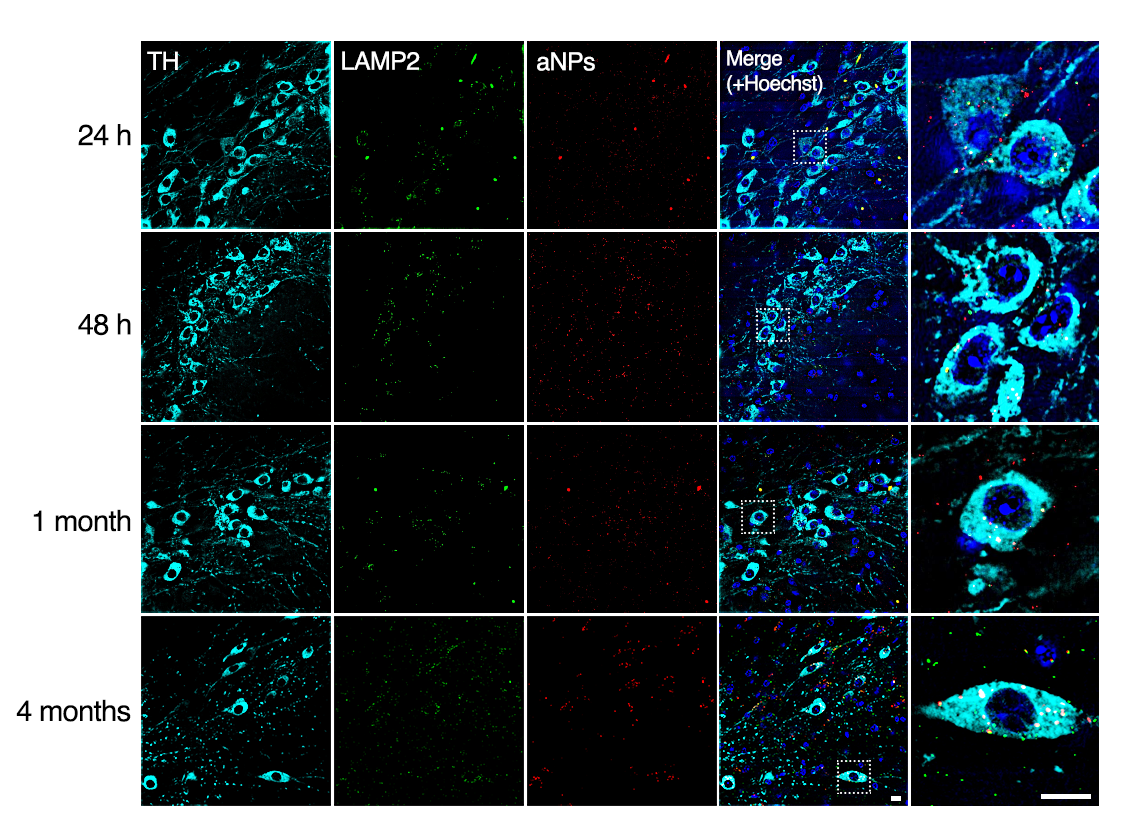


**Figure S3. Acidic nanoparticles are internalized into lysosomes in the nigral dopaminergic neurons of mice.** Representative immunofluorescent images of lysosomal marker LAMP2 *(green)* and Nile red-loaded aNPs *(red)* in Tyrosine Hydroxylase (TH)-positive neurons *(cyan)* of the substantia nigra of mice at 24h, 48h, 1- and 4-month post-injection. Right panels represent insets in a high magnification picture of TH-positive neurons present in the white square. Scale bar: 10µm.


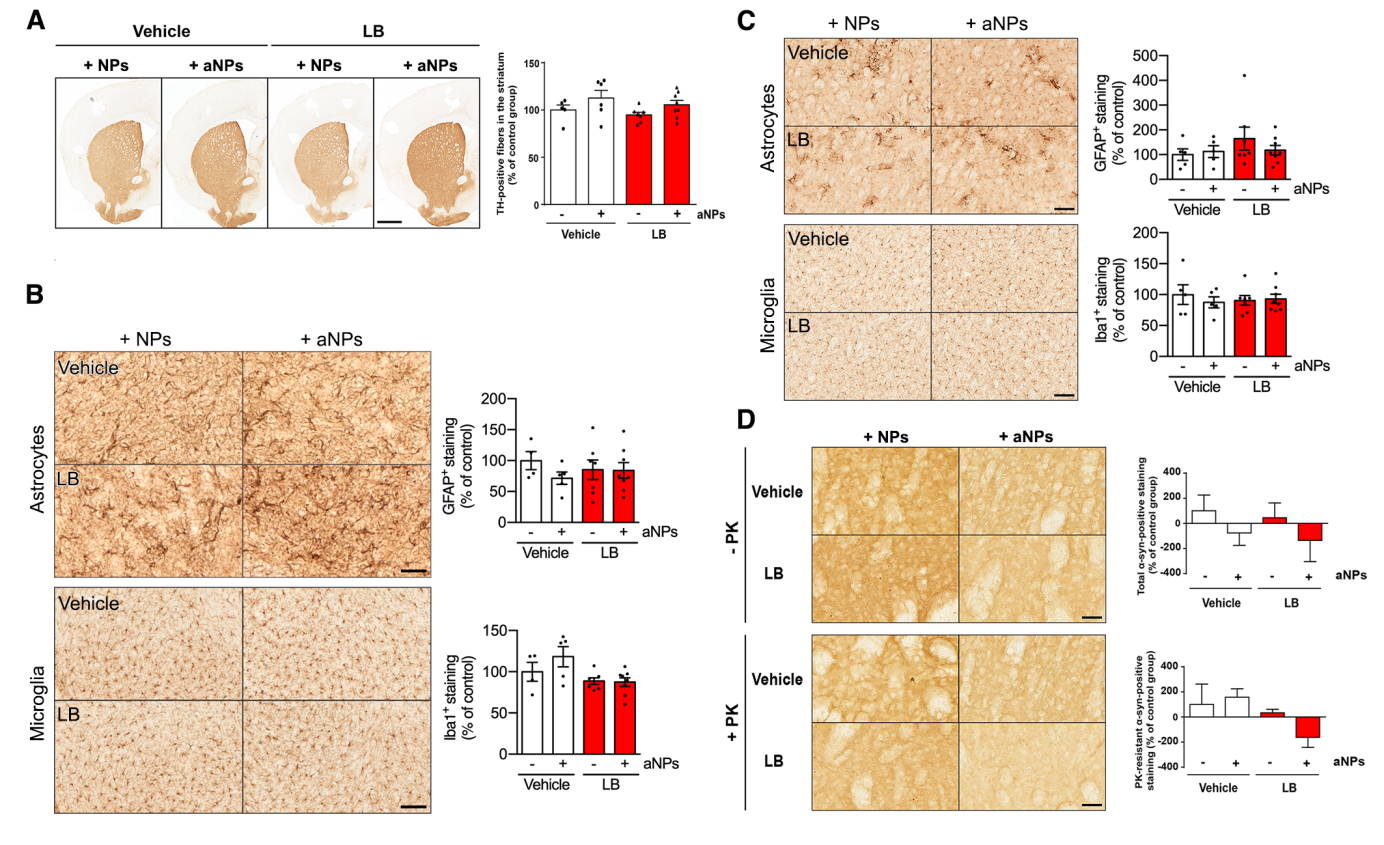


**Figure S4. Acidic nanoparticles do not induce striatal changes nor inflammatory reaction after intranigral injection in mice. (A)** Representative images *(left)* and quantification *(right)* of Tyrosine Hydroxylase (TH) immunostaining measured by optical density in the striatum of control (Vehicle) and LB-injected mice four months after non-acidic nanoparticles (NPs) or acidic nanoparticles (aNPs) injections. Scale bar: 1mm. **(B)** Representative images (*left*) and quantification (*right*) of GFAP/S100-positive astrocytic *(up)* or Iba1-positive microglial cells *(bottom)* immunostaining in the substantia nigra (SN) of control (Vehicle) and LB-injected mice 4-months after injection of non-acidic nanoparticles (NPs) or acidic nanoparticles (aNPs). Scale bar: 200µm. (**C)** Representative images (*left*) and quantification (*right*) of GFAP/S100-positive astrocytic *(up)* or Iba1-positive microglial cells *(bottom)* in the striatum of control (Vehicle) and LB-injected mice four months after non-acidic nanoparticles (NPs) or acidic nanoparticles (aNPs) injections. (**D)** Representative images (*left*) and quantification (*right*) of α-synuclein immunostaining without (*up*) or with (*bottom*) proteinase K (PK) treatment using syn1 antibody in the striatum of control (Vehicle) and LB-injected mice four months after non-acidic nanoparticles (NPs) or acidic nanoparticles (aNPs) injections. Scale bar: 200µm. All panels: n = 5-8 per group. All data are expressed as mean ± SEM. Two-way ANOVA and Tukey’s post-hoc test. [**(A)** F(1,22)=0.03407, p=0.8552 ; **(B)** *GFAP* F(1,20)= 0.8539, p= 0.3665 / *Iba1* F(1,20)= 1.552, p= 0.2272 **(C)** *GFAP* F(1,21)= 0.8191 , p= 0.3757 / *Iba1* F(1,21)= 0.5554, p= 0.4644 ; **(D)** *Top* F(1,17)= 0.0001660, p= 0.9899 / *Bottom* F(1,16)= 1.536, p= 0.2331].


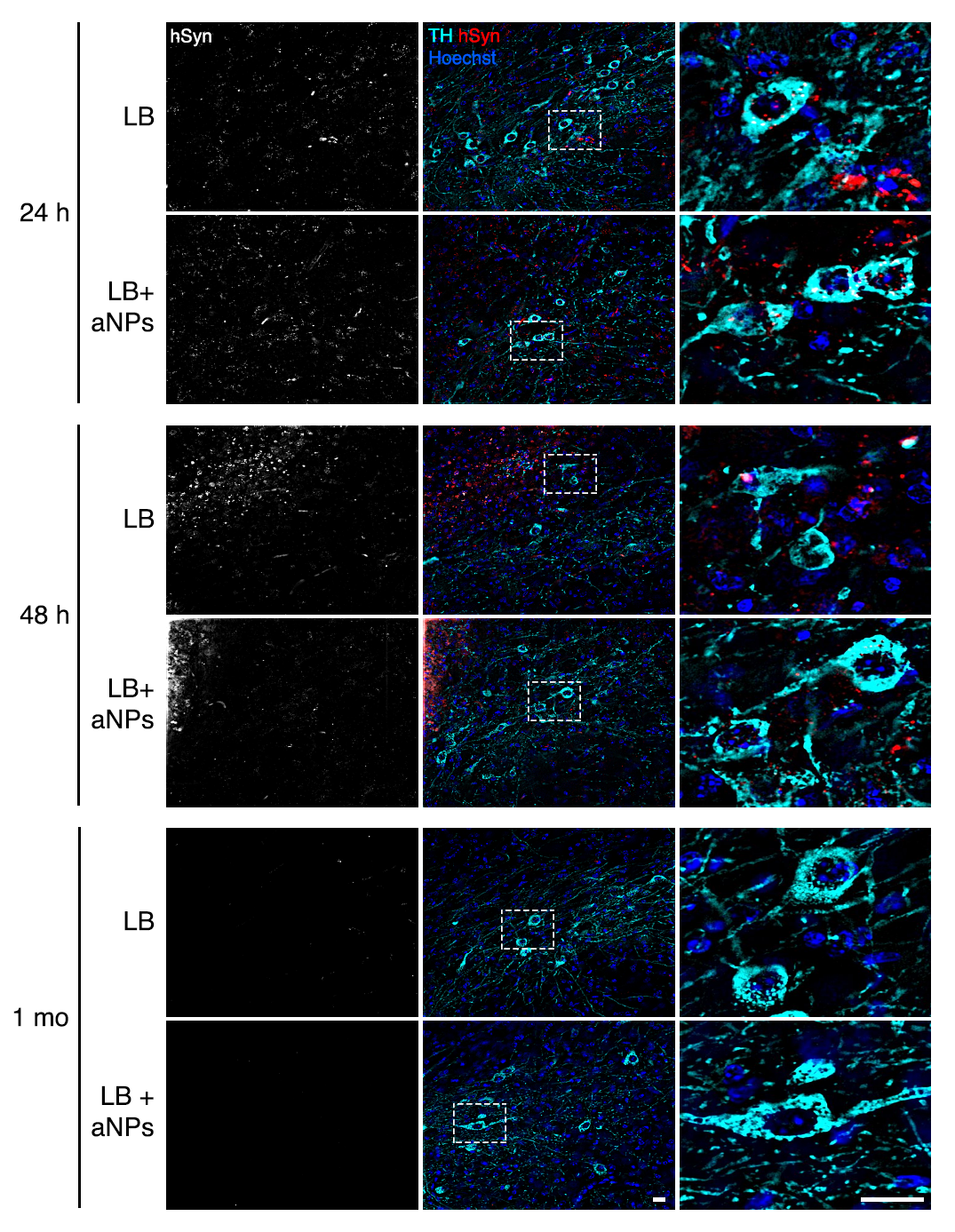


**Fig. S5. Acidic nanoparticles do not prevent uptake of injected human α-synuclein inoculum.** Representative immunofluorescent images of the human α-synuclein immunostaining *(red)* using Syn211 antibody in the Tyrosine Hydroxylase (TH)-positive neurons *(cyan)* of the substantia nigra of LB-injected and LB+aNPs-injected mice at 24h, 48h and 1-month post-injection. White squares were represented as insets in a high magnification picture. Scale bar: 10µm.

**
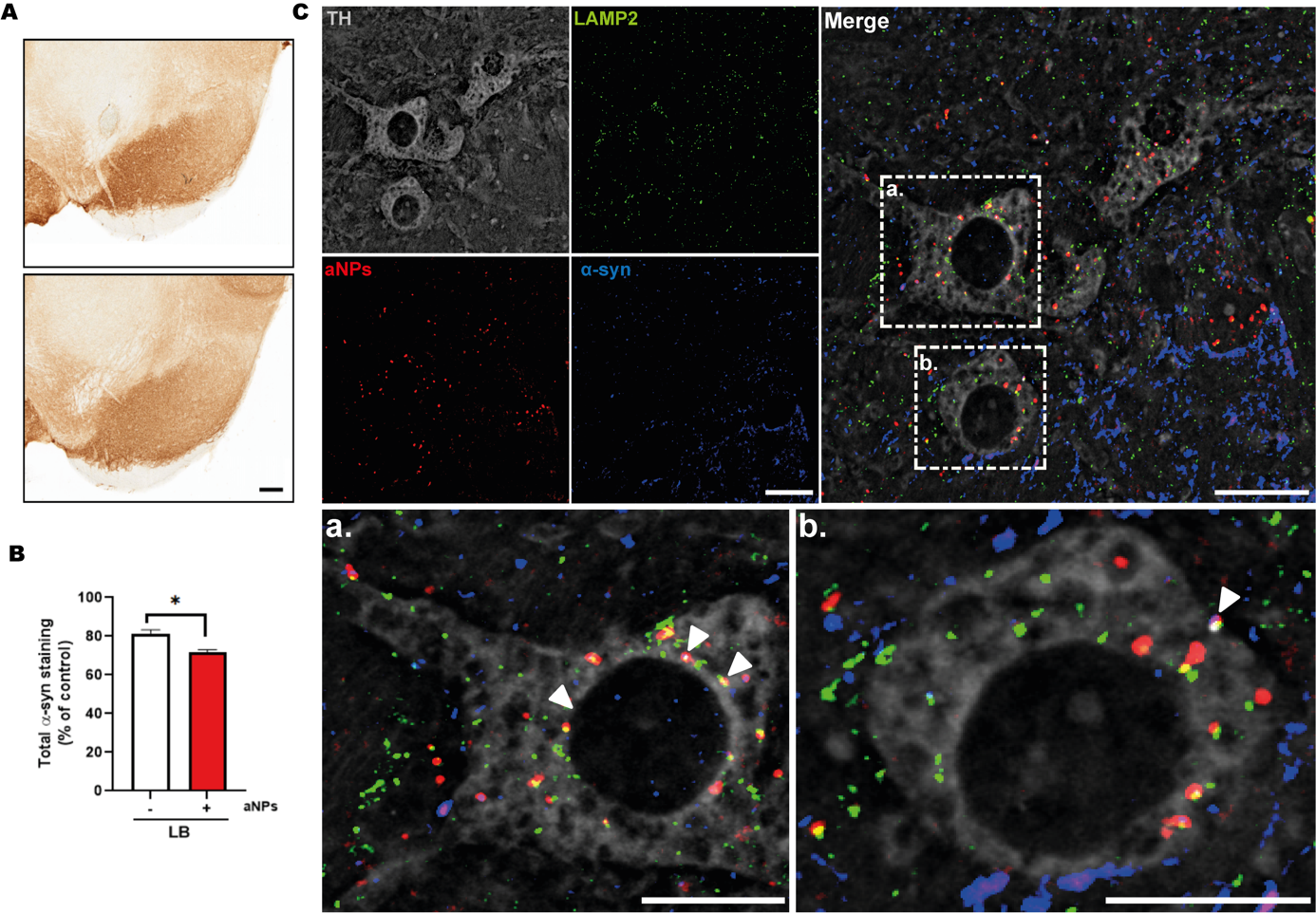
**

**Fig. S6. Acidic nanoparticles prevent synucleinopathy induction by taking up α-synuclein in lysosomes. (A-B)** Representative images (**A**) and quantification (**B**) of murine α-synuclein immunostaining using D37A6 antibody measured by optical density in the substantia nigra of LB-injected mice 1-month after non-acidic nanoparticles NPs (-) or acidic nanoparticles aNPs (+) injections. Scale bar: 200µm. Data are expressed as mean ± SEM. n = 3 per group. Unpaired t-test was performed, p=0.0143. **(C)** Representative immunofluorescent images showing the co-presence of the endogenous α-synuclein using D37A6 antibody *(blue)* with aNP-positive lysosome (Nile red-loaded aNPs, *red*; lysosomal marker LAMP2, *green*) in Tyrosine Hydroxylase (TH)-positive neurons *(grey)* of the substantia nigra of mice at 1month post-injection. Scale bar: 20µm. Panels *a.* and *b.* represent insets in a high magnification picture of TH-positive neurons present in the white square. Scale bar: 10μm. White arrows show the co-presence of the four markers (white pixels).

**
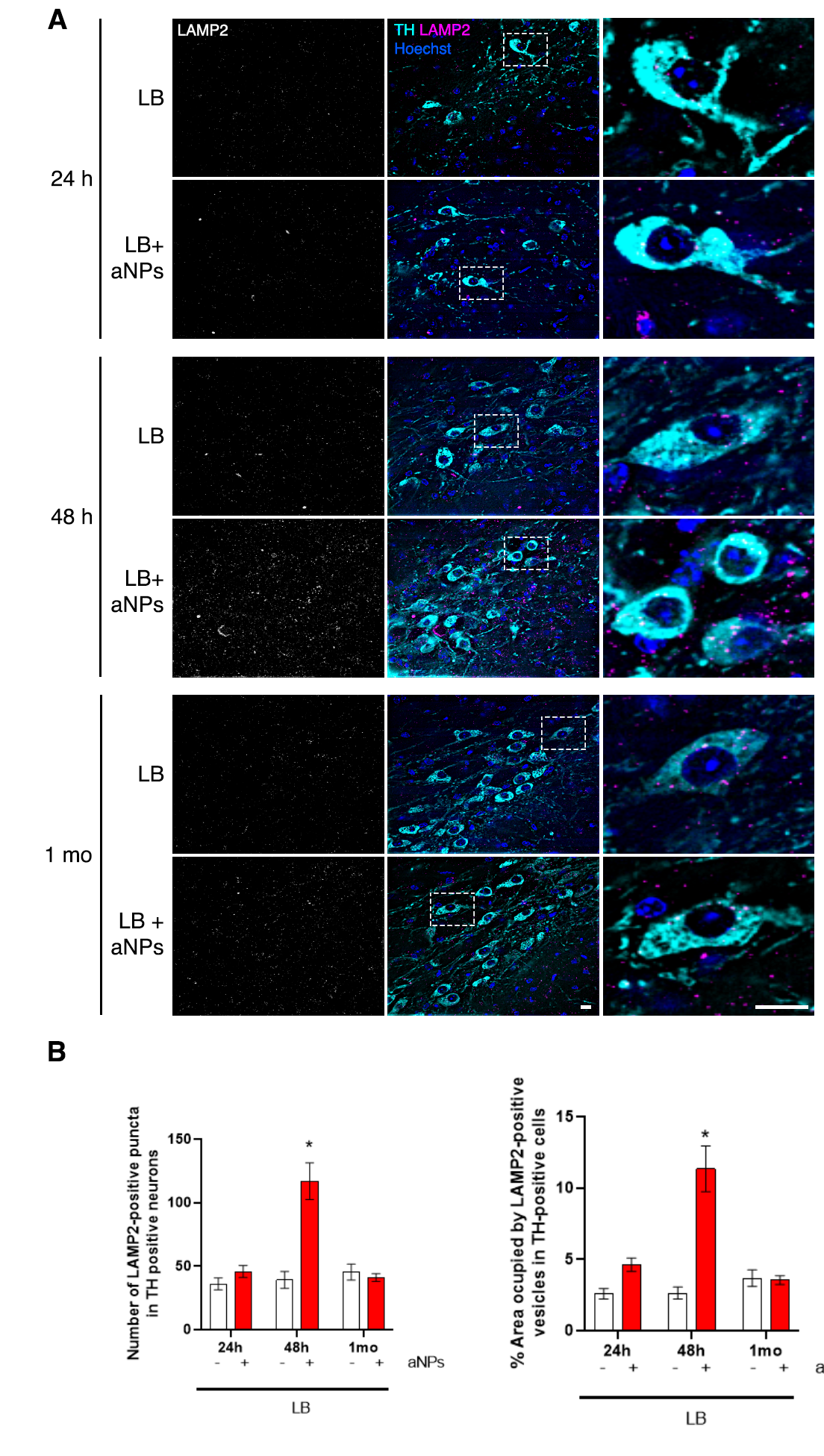
**

**Fig. S7. Acidic nanoparticles induce lysosomal biogenesis. (A)** Representative immunofluorescent images of the lysosomal marker LAMP2 *(purple)* in the Tyrosine Hydroxylase (TH)-positive neurons *(cyan)* of the substantia nigra of LB-injected mice at 24h, 48h and 1-month after non-acidic nanoparticles (LB) or acidic nanoparticles (LB+aNPs) injections. Selected white squares were represented as insets in a high magnification picture. Scale bar: 10µm. **(B)** Quantification of the number of *(left)* and percentage of area occupied by *(right)* LAMP2-positive puncta present in the Tyrosine Hydroxylase (TH)-positive neurons of the substantia nigra of LB-injected mice at 24h, 48h and 1-month after non-acidic nanoparticles NPs (-) or acidic nanoparticles aNPs (+) injections. A total of 7-41 neurons were assessed per group. n=2-3 animals per group. Data are expressed as mean ± SEM. Two-way ANOVA and Tukey’s post-hoc test [**(B)** *left* F(2,242)= 19.77, p< 0.0001 / *right* F(2,242)= 21.90, p< 0.0001]


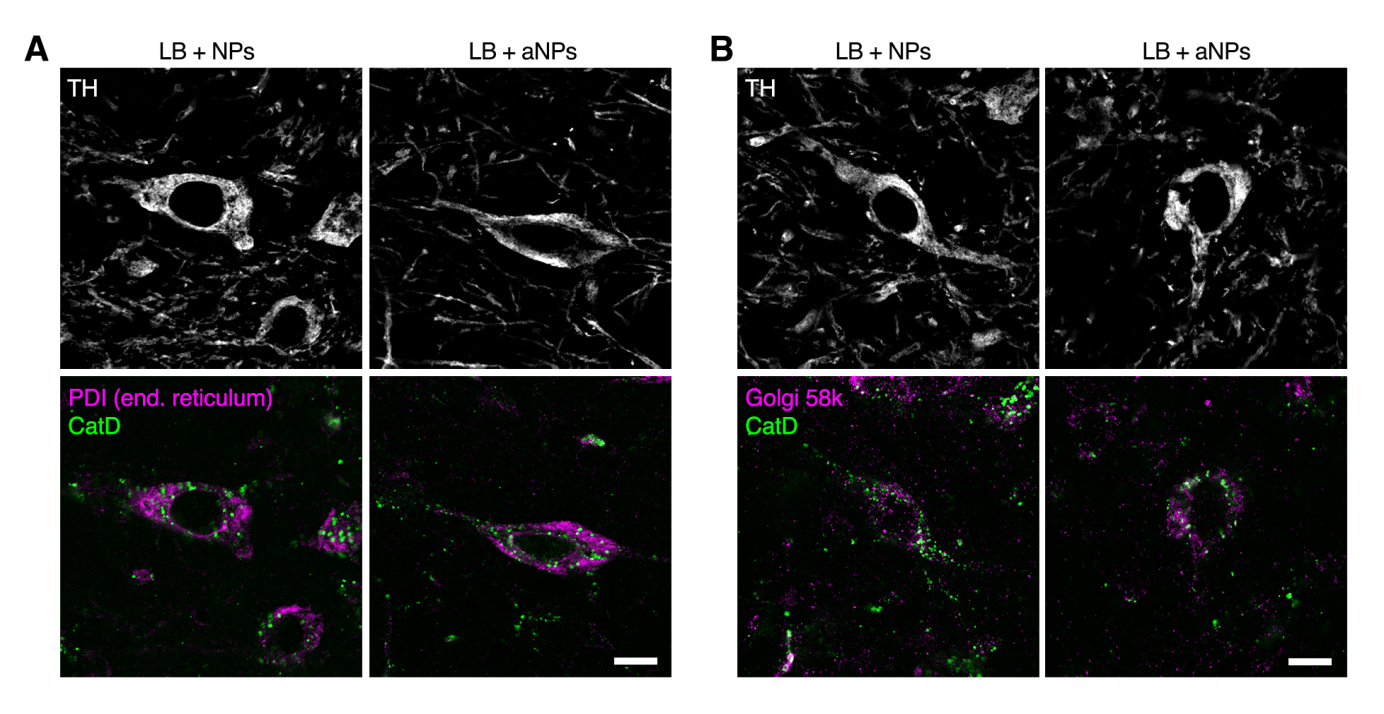


**Figure S8. Lysosomal protease Cathepsin D is not localized in the endoplasmic reticulum or the Golgi apparatus of the dopaminergic neurons in the SN of LB-injected mice after aNPs administration. (A)** Representative confocal micrographs of triple immunostaining for the dopaminergic marker tyrosine hydroxylase (TH), the endoplasmic reticulum marker (PDI), and the lysosomal protease Cathepsin D (CatD) in the substantia nigra (SN) of LB-injected mice at 4-months after non-acidic nanoparticles (NPs) or acidic nanoparticles (aNPs) injections. **(B)** Representative confocal micrographs of triple immunostaining for the dopaminergic marker tyrosine hydroxylase (TH), the Golgi marker (58K protein) and the lysosomal protease Cathepsin D (CatD), in the substantia nigra (SN) of LB-injected mice four months after non-acidic nanoparticles (NPs) or acidic nanoparticles (aNPs) injections. Scale bar = 10 µm.

**Supplementary Movie 1**

**Description:** aNPs (*red*) are found within LAMP1-positive puncta (*green*).

**Supplementary Table 1.** Raw data that served for the study analyses for all histological and biochemical approaches.
